# Secular trends and sociodemographic disparities in physical activity among adults in eleven African countries: WHO STEPS 2003–2020

**DOI:** 10.1186/s12966-024-01675-7

**Published:** 2024-10-30

**Authors:** Adewale L. Oyeyemi, Raphael H.O. Araujo, Umar A. Hassan, Edward Ofori, Chad Stecher, André O. Werneck

**Affiliations:** 1 College of Health Solutions, Arizona State University, 85004 Phoenix, AZ USA; 2 Graduation Program in Health Sciences, Londrina State University, 86057-970 Londrina, Brazil; 3 School of Public Health, The University of Queensland, 4006 Brisbane, QLD Australia; 4 Center for Epidemiological Research in Nutrition and Health, Department of Nutrition, School of Public Health, Universidade de São Paulo (USP), São Paulo, SP Brazil

**Keywords:** Prevalence, Monitoring, Surveillance, Leisure-time physical activity, Transport physical activity, Occupation physical activity

## Abstract

**Background:**

Mortality from physical inactivity-related non-communicable diseases (NCDs) is projected to surpass deaths from communicable diseases by 2030 in Africa. Monitoring physical activity (PA) is important for planning public health interventions addressing NCDS and planetary health, but there is a dearth of evidence on PA trends in Africa. This study explored the secular trends in overall and domains of PA (leisure, occupation, and transport), and examined the gender, age, and education disparities in PA trends across African countries.

**Methods:**

We utilized data from the STEPwise approach to NCDs risk factor surveillance in eleven African countries (Algeria, Benin, Botswana, Cabo Verde, Eritrea, Eswatini, Malawi, Mali, Central Africa Republic, Sao Tome and Principe, and Zambia) with at least two surveys conducted between 2003/2010 (first-wave) and 2010/2020 (second-waves). A total of 29,282 and 40,147 adults (18–69 years) in the first and second waves, respectively, completed PA interviews using the Global Physical Activity Questionnaire. Gender, age, and education status were self-reported. Weighted individual-country PA prevalence and 95% confidence interval (95%CI) were obtained. Random-effect meta-analysis was conducted to assess pooled estimates of PA trends across countries. Gender, age, and education disparities in PA trends were also investigated.

**Results:**

Country-specific results showed significant upward trends in total PA in eight countries. Seven countries showed significant increasing trends in some leisure-time PA (2.0% − 13.9% increase) and ≥ 150 min/week transport PA (4.0% − 24.5% increase), while five countries recorded significant increasing trends in occupational PA (6.6% − 56.9% increase). Gender, age and education disparities in meeting the WHO PA guidelines remained relatively stable over time, but disparities in leisure, transport and occupational PA increased in most countries.

**Conclusions:**

The prevalence of overall PA among African adults has marginally increased over 17 years. There are still many adults, especially women and people with lower education, not doing well in domain specific PA. Policy and environmental interventions are needed to improve PA and to reduce gender, age, and education disparities in leisure, transport, and occupational PA in African countries.

**Supplementary Information:**

The online version contains supplementary material available at 10.1186/s12966-024-01675-7.

## Introduction

Noncommunicable diseases (NCDs), including stroke, heart diseases, cancers, chronic respiratory diseases, and diabetes, are the leading cause of death worldwide and are responsible for 41 million (74%) deaths globally in 2023 [1]. Low- and middle-income countries, including Africa, experience over 86% of the premature deaths from NCDs annually [1]. In Africa, the proportion of deaths due to NCDs ranged from 36–88% in 2019, and these rates are projected to increase and exceed deaths due to communicable diseases by 2030. These trends threaten progress towards the 2030 Agenda for Sustainable Development in the region [2, 3]. To effectively address these changing needs and control the rising prevalence of NCDs, countries in Africa need improved epidemiological data on NCDs risk and resilience factors [3, 4].

Physical inactivity is a major risk factor and one of the most important preventable causes of morbidity and deaths due to NCDs [5]. Beyond its benefits on NCDs, physical activity (PA) has multiple environmental co-benefits, including the potential for protection against climate change and improving planetary health [6, 7]. To address the rising challenge of physical inactivity, the World Health Organization (WHO) launched a global action plan to reduce physical inactivity by a 15% by 2030 [8]. However, achieving these goals in Africa is hampered by a lack of PA research and surveillance data [9]. National surveillance and monitoring of physical (in)activity epidemiology are not just needed to address chronic conditions and NCDs in Africa [10], but are crucial for policies and programs to mitigate infectious diseases [11, 12] and climate change consequences such as air pollution and extreme weather in the region countries [13]. Regular population surveillance of PA in a standardized manner contributes significantly to strengthening national, regional, and global comparisons of PA behaviors [14, 15]. While national and international PA surveillance using standardized methods has increased in recent years, few studies have used these data to investigate trends in the prevalence of PA across African countries.

Through the WHO Stepwise Approach to Chronic Disease Risk Surveillance (STEPS) initiative, the number of African countries with standardized surveillance of PA at the national or subnational level has increased from 22 in 2002, when the first STEPS was conducted, to 41 in 2019 [16–18]. This initiative demonstrated that standardized and validated questionnaires are feasible and effective for tracking the prevalence of PA in Africa. Guthold and colleagues, in their analysis of the WHO STEPS study in 22 African countries, found 83.8% of men and 75.7% of women met the WHO PA guidelines in the 2003 and 2009 waves of the STEPS. This research also found occupational PA and transport PA contributed most to overall PA, while leisure-time PA was the least performed PA type [18]. Also, a recent analysis of 10 population-based surveys of adults in nine African countries using similar survey methods as the WHO STEPS project found lower leisure-time PA compared with occupational PA and transport PA [19]. Further, women, older adults, less educated, self-employed, and rural populations were less likely to participate in leisure-time PA across the nine countries surveyed [19]. These studies also observed occupational PA and transport PA as the dominant forms of PA in Africa [18–22]. While this research represents important progress made in African PA surveillance and highlights the feasibility of using similar methods for cross-country comparisons of PA behaviors, consistent data for secular and temporal trends of PA across African countries remain scarce.

The study by Guthold and colleagues provides the most comprehensive global estimates of the prevalence and trends of PA [17]. Their study included forty-one Sub-Saharan African countries, but only four (Benin, Botswana, Seychelles, and South Africa) had at least two comparable surveys from different years (2003–2015) using the same questionnaire to allow for meaningful trend analyses. Since the study of Guthold et al in 2018 [17], there are now 11 African countries with the same STEPS PA survey conducted at two time points. More recently, Strain and colleagues in 2024 updated the global estimates and time trends of insufficient PA (from 2000 to 2022) for 197 world countries, including 20 sub-Saharan African countries with at least two eligible PA surveys at different time point [23]. However, both Guthold et al [17] and Strain et al [23] estimated only the trends of meeting the guidelines for overall (total) moderate-to-vigorous PA (MVPA). Yet, important gaps remain in our understanding of the trends in different domains of PA as well as in disparities in PA trends across sociodemographic subgroups. Investigating trends in domains of PA is particularly important because it can reveal socioeconomic inequities and different opportunities and access to PA within and between populations in Africa. Also, different domains of PA can be differently associated with NCDs. For instance, while previous research found leisure-time PA is consistently associated with lower risks for cardiovascular diseases and mortality, other domains, such as high occupational PA, are important risk factors for poor cardiovascular health and early mortality [24, 25].

For effective public health interventions, it is also important for PA surveillance research to examine trends in PA levels (i.e., identifying those with no or some PA) rather than focusing only on those meeting the WHO threshold for sufficient MVPA [26]. The WHO PA guidelines advocate that some PA is better than none for those not meeting the recommended MVPA threshold [27]. Also, doing only some PA can be an indicator of a lack of access or opportunity to practice PA, especially during leisure time [28]. Moreover, the use of different thresholds to monitor PA could provide a more nuanced interpretation of the surveillance data and help present a more comprehensive view of the progress and trends of PA in regions with lower surveillance coverage [26]. In the present study, we use nationally-representative data from 11 African countries participating in the WHO STEPS to: 1) estimate the trends in total PA minutes, reaching at least 150 minutes per week, or performing any PA (at least 1 minutes/week) by domains (i.e., leisure-time, occupation, transport, and total PA), and 2) examine the sociodemographic (i.e., gender, age and education) disparities in PA trends over time.

## Methods

### Sample

We utilized data from the WHO STEPS initiatives. The STEPS framework and data collection methods have been fully described elsewhere [16, 29]. Briefly, STEPS is a standardized framework for countries to monitor NCD risk factors by conducting cross-sectional, nationally representative surveys, using standardized questionnaires, and biochemical measurements among the adult population (18–69 years). The STEPS surveys are coordinated by authorities from each country. Although it uses standardized procedures, STEPS is also flexible for the inclusion of some variables, providing varying information based on each country's specific strategies and available resources. The STEPS framework is divided into three levels of variables. The first level includes general sociodemographic and behavioral information through self-report and physical measurements, including body weight, stature, waist circumference, and blood pressure. The second and third levels include blood-derived risk factors, such as fasting glucose and urinary sodium. Within each level, there is a core questionnaire and additional items that can be included at the discretion of each country. The sampling process can vary according to the characteristics of each country, but usually, a multistage cluster sampling is conducted. Due to the multistage sampling design nature of the STEPS surveys, sampling weights are estimated after the completion of each survey to maintain the representativeness of the sample.

Considering our aim of analyzing time-trends, we used data from African countries that conducted at least two STEPS surveys from 2003 to 2020. Therefore, we included eleven African countries (Algeria, Benin, Botswana, Cabo Verde, Eritrea, Eswatini, Malawi, Mali, Central Africa Republic, Sao Tome and Principe, and Zambia) that conducted two STEPS surveys between 2003–2010 (the first wave) and 2010–2020 (the second wave) depending on the country periods of data collection (Supplementary Table 1). There were no countries with three waves of nationally representative data.

## Physical activity

PA was assessed using the Global Physical Activity Questionnaire (GPAQ) [30]. The questionnaire includes questions about the frequency and duration of PA practiced in different domains, such as leisure-time, occupational, and transport. We estimated an indicator of total PA/week by combining the different domains and identified those meeting the WHO 2020 recommendations for moderate-to-vigorous aerobic activity: at least 150 min/week of moderate-intensity activity, 75min/week of vigorous-intensity activity, or an equivalent combination [31]. We also created an indicator of sufficient PA (herein refers as at least 150min/week) for each domain (i.e. leisure-time, occupation, and transport), as well as another indicator of some (≥ 1min/week) PA for each domain, considering their complementation for the estimation of PA inequalities [26]. The GPAQ has been shown to have acceptable evidence of reliability (Kappa 0.67 to 0.73; Spearman's rho 0.67 to 0.81) across nine countries, including two African countries [32]. Its validity, as tested by correlations with pedometers (median rs = .31), was comparable to other self-reports [32].

## Gender, age, and education

Gender was assessed through the self-report of biological sex (male/female). In this paper, we used the term ‘gender’ because evidence indicates that the PA gap between men and women seems more related to a social construction (gender) than a biological one (sex) [33–35]. Chronological age was self-reported and classified as 1) 18-34y; 2) 35-49y; 3) + 50y. Education was assessed through the self-reported highest academic qualification. We classified education as “no formal education”, “less than high school” or “high school or more.”

## Statistical procedures

All our analyzes considered the specific sampling weights for each survey. The sample was described using absolute frequencies. Weighted relative frequencies and their respective 95% confidence interval (95% CI) were used to describe the country-specific prevalence of PA in each wave of data collection, considering each country's gender, age group, and education. After the calculation of the prevalences in each survey, we used a random-effect meta-analysis of proportion to obtain the pooled estimates of PA trends across countries, considering each survey (year) in each country as an independent survey entry. Meta-analysis was conducted through the “metaprop” command in Stata 18.0. Gender, age, and education disparities were assessed by estimating the absolute gap between the extreme categories in percentage points (p.p.). We identified changes as significant when there was no overlap in the 95% confidence intervals of estimates between survey years. All analyses were conducted using Stata 18.0 (StataCorp., College Station, TX, USA).

## Results

We included data from eleven countries with two surveys, comprising 29,282 participants in the first wave and 40,147 participants in the second wave. Details of the included countries' sample, STEPS survey years, coverage, and country-level socioeconomic and human development indices are in Supplementary Table 1. The majority of the countries (n = 9) were categorized as either low-income or lower-middle-income countries during the periods in which the STEPS surveys were conducted. Results for the trends and prevalence of different domains of PA in the 11 countries are shown in Table 1.

**Table 1:**
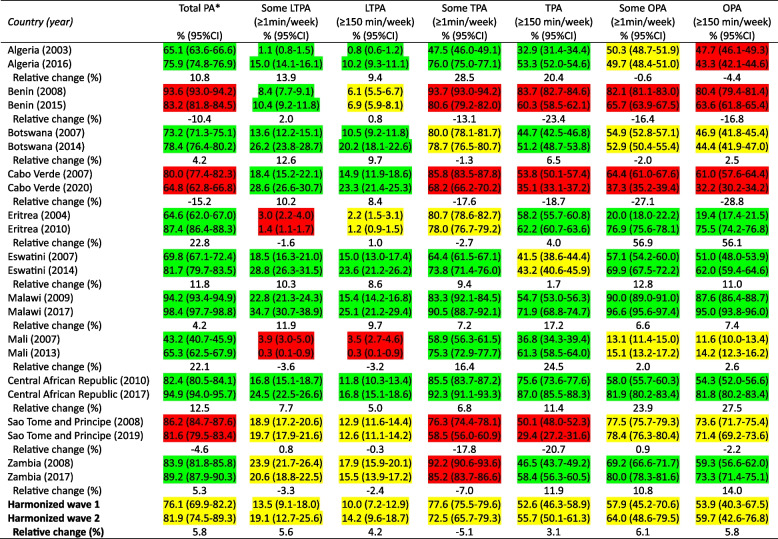
Prevalence of different domains of physical activity according to country and wave

*PA* physical activity, *LTPA* leisure-time physical activity *TPA* transport physical activity, *OPA* occupational physical activity, Color legend: Green, significant increased trend; Red, significant decreased trend; Yellow, not significant trend

^*^Using WHO 2020 guidelines on physical activity of at least 75min/week of vigorous physical activity or 150min/week of the combination of moderate and vigorous physical activity

Country-specific analyses showed eight (Algeria, Botswana, Eritrea, Eswatini, Malawi, Mali, Central African Republic, and Zambia) of eleven countries had a significant upward trend in the prevalence of compliance with the WHO total PA guidelines, while three countries decreased their prevalence (Benin, Cabo Verde, and Sao Tome and Principe). Although being the domain with the lowest prevalence of PA, seven countries (Algeria, Benin, Botswana, Cabo Verde, Eswatini, Malawi, and Central African Republic) recorded increasing trends in the prevalence of some leisure-time PA (range = 2.0–13.9% increase), while two reduced (Eritrea and Mali, range= -3.6% to -1.6% decrease). Six countries each recorded increasing trends in the prevalence of ≥ 150 min/week of leisure-time PA (range = 5.0–9.7% increase), while it was reduced only in Mali (-3.2% decrease). Similarly, seven countries recorded increasing trends in the prevalence of ≥ 150 min/week of transport PA (range = 4.0–24.5% increase). However, four countries (Benin, Cabo Verde, Sao Tome and Principe, and Zambia) also recorded decreasing trends in the prevalence of some transport PA (-7.0% to -17.8% decrease). Five countries each recorded increasing trends in the prevalence of some occupational PA and ≥ 150 min/week of occupational PA (Eritrea, Eswatini, Malawi, Central African Republic, and Zambia; range = 6.6% − 56.9% increase) (Table 1).

**Fig. 1:**
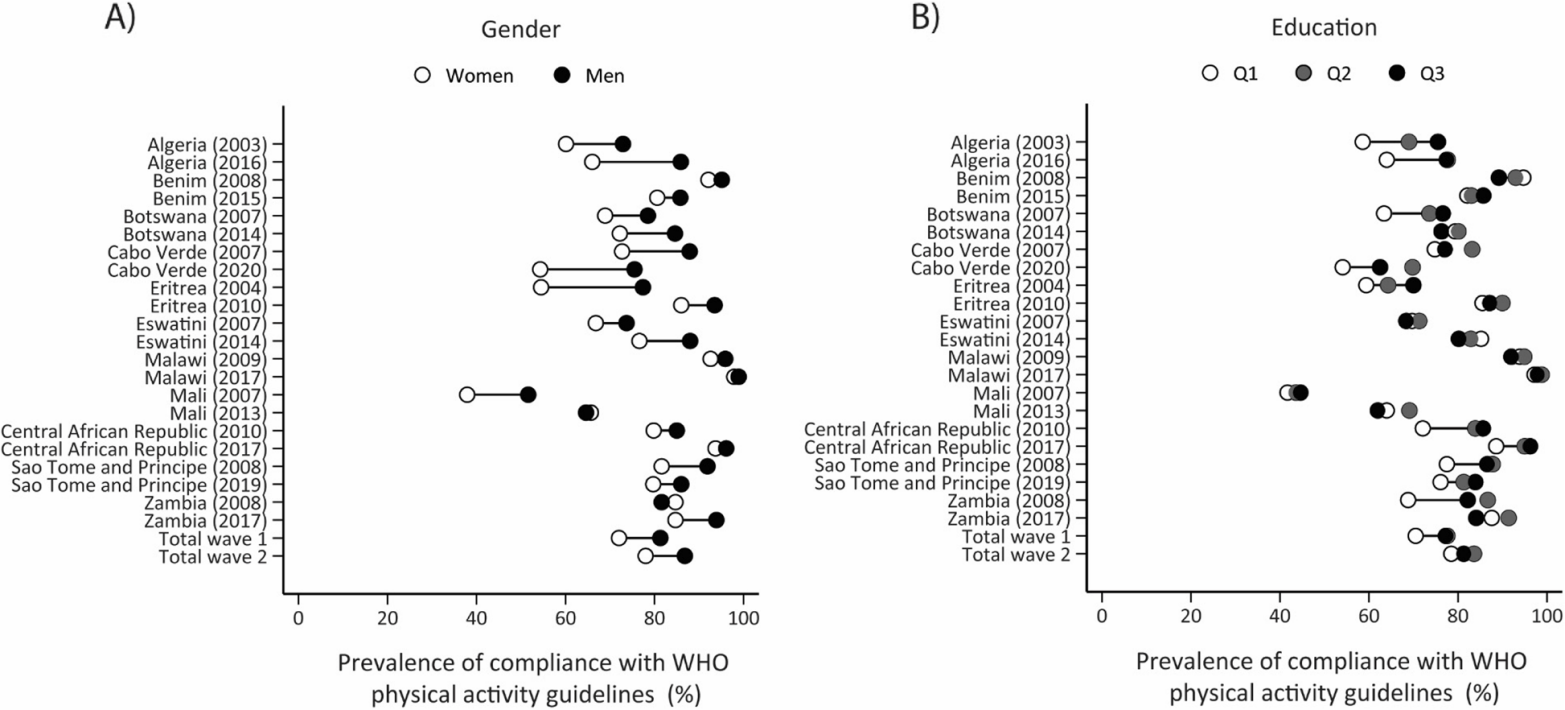
Equiplots illustrating gender and education inequalities, along with trends in compliance with the WHO 2020 physical activity guidelines

Figure 1 and Supplementary Tables 2 and 15 present the trends of gender, education, and age disparities in PA practice for compliance with the WHO PA guidelines. Although gender disparities in PA practice increased in six countries (Algeria, Benin, Botswana, Eswatini, and Sao Tome and Principe), they decreased in the other five countries, resulting in relative stability in gender disparities over time. Similarly, the education and age disparities in meeting the WHO PA guidelines were relatively stable over time for most of the countries.

**Fig. 2:**
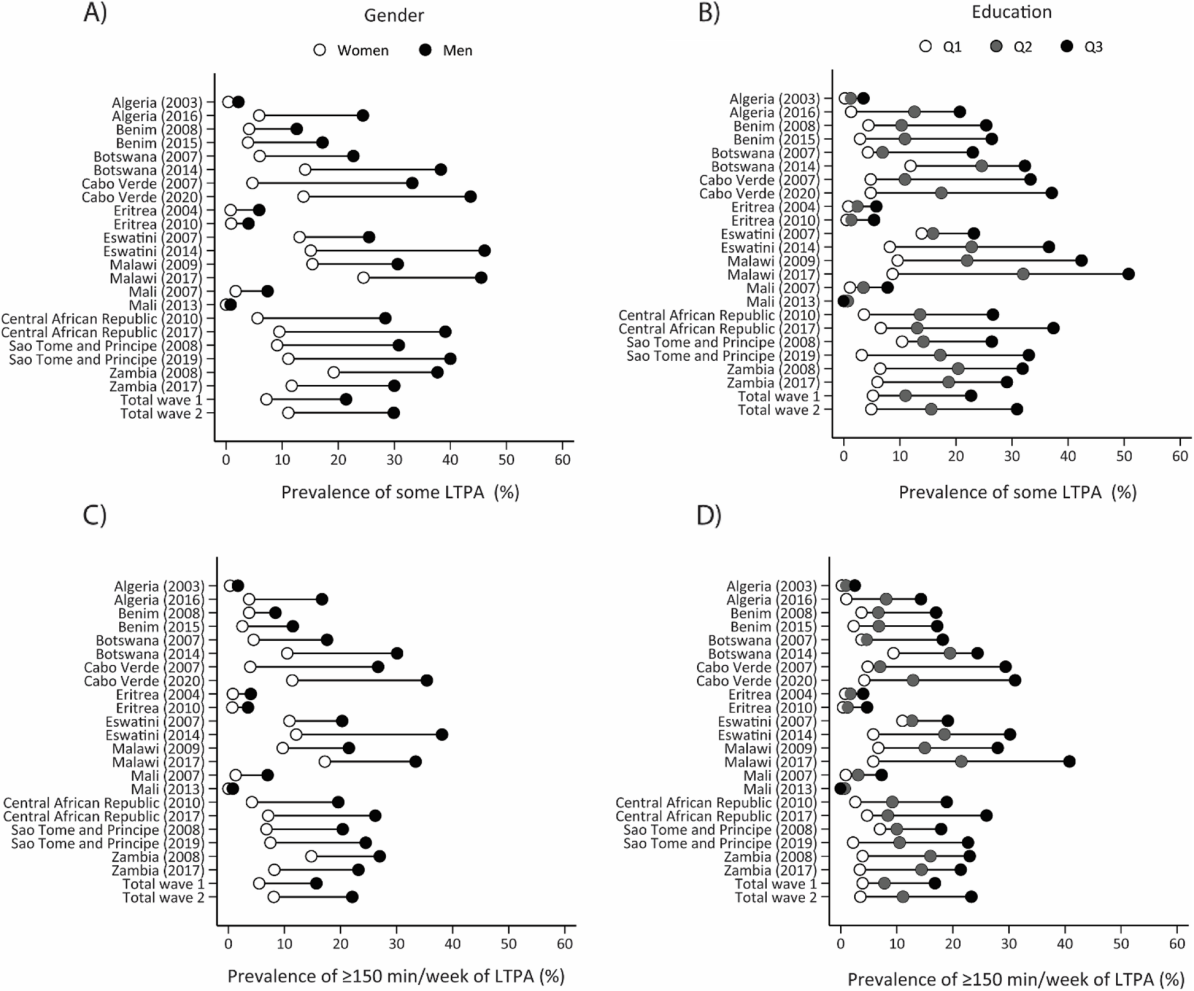
Equiplots illustrating gender and education inequalities, along with trends in leisure-time physical activity. Note. LTPA, leisure-time physical activity

Figure 2 and Supplementary Tables 3, 4, 9, and 10 present the trends and disparities in leisure-time PA. In general, there was an increasing trend in gender, age, and education disparities for both practicing some leisure-time PA (gender gaps of -14.2 in the first wave and − 18.8 in the second wave) and ≥ 150 min/week (gender gaps of -10.2 in the first wave and − 14.0 in the second wave) over time, with men, younger adults, and people with higher education presenting higher prevalence. For instance, while the group with the highest education increased the prevalence of some leisure-time PA (from 22.7%, 95%CI: 15.8–29.5 in wave 1 to 30.9%, 95%CI: 22.5–39.2% in wave 2), the group with no formal education presented a slight reduction (from 5.2%, 95%: 2.1–8.3 in wave 1 to 4.9%, 95%CI: 3.8-6.0 in wave 2). Algeria, Central African Republic, Eswatini, Malawi, and Sao Tome and Principe were the countries in which gender, age, and education disparities in leisure PA increased the most.

**Fig. 3:**
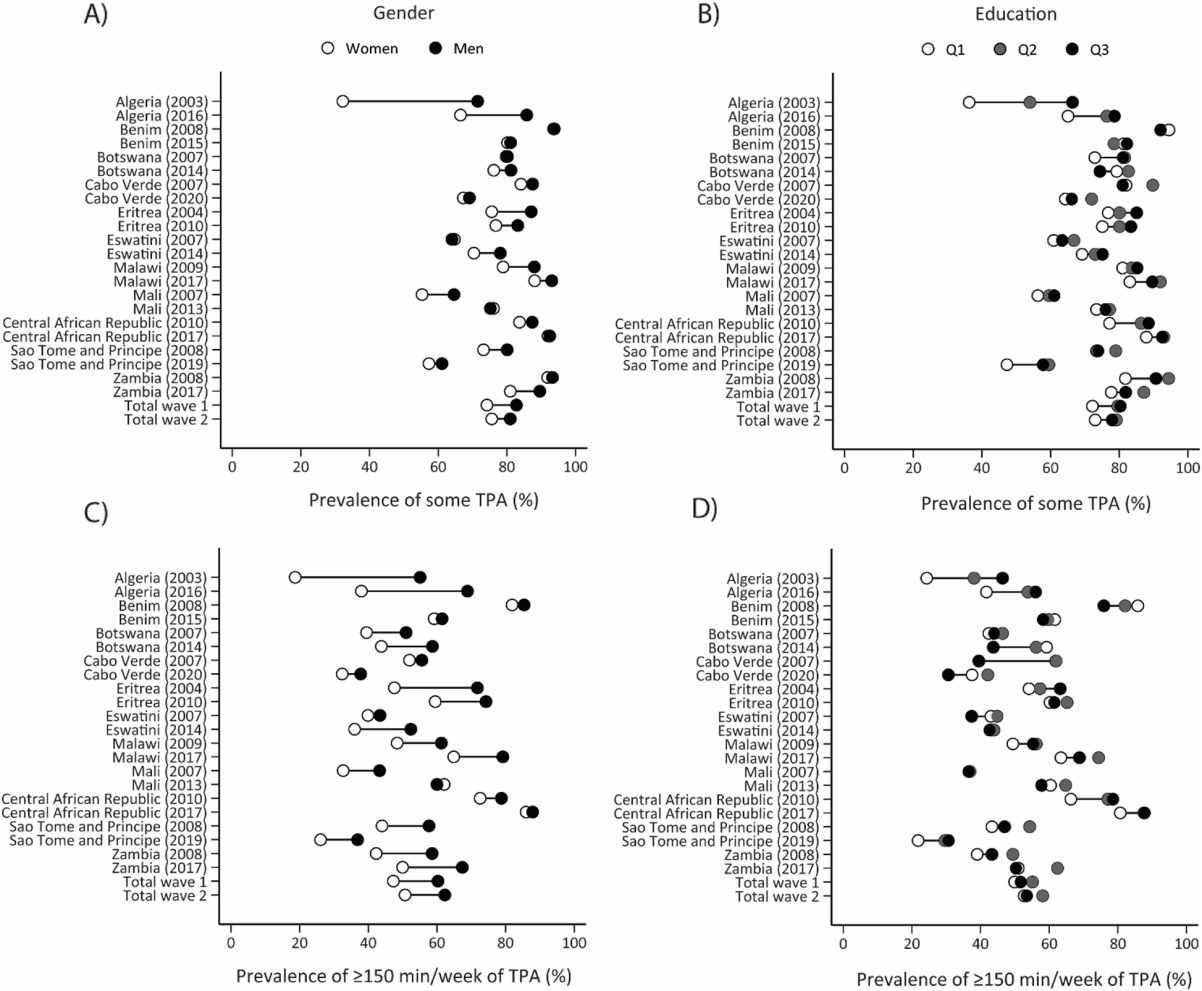
Equiplots illustrating gender and education inequalities, along with trends in transport physical activity. Note. TPA, transport physical activity

There were observable disparities in transport PA, with men consistently having a higher prevalence of some and ≥ 150 min/week of transport PA, while people with higher education presented a slightly higher prevalence of some transport PA but a lower prevalence of ≥ 150 min/week transport PA. However, the gender and education disparities in transport PA tend to decline over time, with gender disparities in the prevalence of some transport PA declining from − 8.6 p.p. in the first wave to -5.4 p.p. in the second wave. Also, education disparities in the prevalence of ≥ 150 min/week transport PA declined from − 1.7 p.p. in the first wave to -0.7 p.p. in the second wave (Fig. 3 and Supplementary Tables 5 and 6). Age disparities were low and relatively consistent over the years (Supplementary tables 11 and 12). Algeria had the largest gender and education disparities in transport PA, while the Central African Republic was where age disparities in transport PA increased the most (Supplementary Tables 5, 6, 11, and 12).

**Fig. 4:**
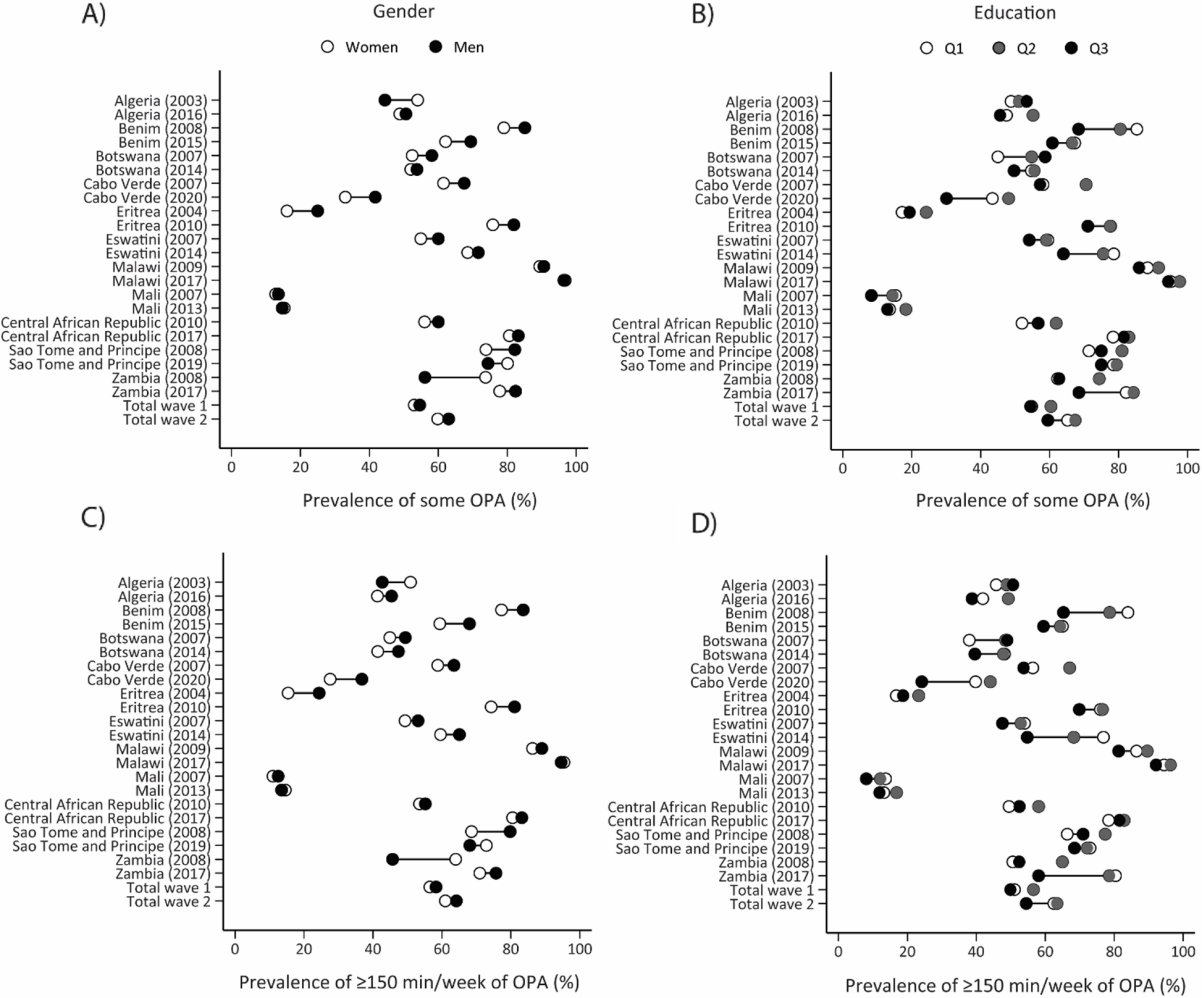
Equiplots illustrating gender and education inequalities, along with trends in occupational physical activity. Note: OPA, occupational physical activity

For occupational PA, the disparity gaps in the prevalence of some occupational PA (-1.6 p.p. in wave 1 vs. -3.2 p.p. in wave 2) and ≥ 150 min/week occupation PA (-1.8 p.p. in wave 1 vs. -3.2 p.p. in wave 2) were higher in men than women, although they were relatively minor and stable. While occupational PA (both thresholds) increased by ~ 11 p.p. among those with no education, the increments among those with high school were around 5 p.p. The disparity gap in occupation PA indicators widens between those with less than high school and those with high school or more education, with a higher prevalence among those with less than high school (Fig. 4 and Supplementary Tables 7 and 8). Age disparities were also small and tended to reduce over time (Supplementary tables 13 and 14). Benin, Botswana, Cabo Verde, Eswatini, Central African Republic, and Zambia were the countries where gender, age and education disparities in occupation PA increased most.

## Discussion

This is, to our knowledge, the first study to examine secular trends across multiple PA domains and thresholds among adults in several African countries. Our analysis of secular trends in PA of African adults covers a varying period in the past 17 years, a period when the burden of NCDs increased to 67% in the African population [4]. Overall, we found marginal upward trends for total PA in meeting the WHO PA guidelines and in at least 150 min/week of leisure-time, transportation, and occupation PA, suggesting steady maintenance in PA of African adults over time. The results also suggested upward trends for participation in some (≥ 1min/week) PA across all domains except for a decrease in some transport PA. Though we found leisure-time PA has increased over time in many countries, it is still the domain with the lowest prevalence of PA, consistent with patterns reported in previous cross-sectional studies among African adults [18–22]. The overall steady maintenance of PA levels across multiple domains is promising and suggests some progress towards meeting the WHO global action plan to reduce physical inactivity by a relative 15% by 2030 [8]. Yet, compared to high-income regions [17, 36], the prevalence and trends of insufficient leisure-time PA remain relatively high in Africa, indicating an urgent need for effective national and regional efforts to improve this domain of PA in Africa. Eliminating barriers to leisure PA participation in Africa can facilitate equitable access to the health and social benefits associated with this domain of PA among African adults [19].

The finding of an overall decrease in some transport PA and not in at least 150 min/week of transport PA and other domains of PA is difficult to explain, especially when the country-level income and human development index for most countries in the region did not witness appreciable changes between survey periods. However, access to cars and the conditions of public transportation may have improved over time in many African countries, and people who previously had no choice but to use active transportation now have the option of choosing other modes of transportation. Still, it may reflect an issue of equitable access and opportunity for recreational-related transport PA (e.g., walking and biking for recreation) in Africa, as inequalities in PA are more detectable when thresholds are set to identify those with no or low (some) PA [26]. Africa is the region with the lowest infrastructure and opportunities to engage in walking and biking in a safe environment [13]. Perhaps these conditions have worsened over time and are more pronounced, especially in the four countries (Benin, Cabo Verde, Sao Tome and Principe, and Zambia) where the largest decline in some transport PA occurred. However, it should be noted that the decline in most PA domains reported in Cabo Verde could be the artifact of the reduction in PA levels witnessed during the COVID-19 pandemic [37], as the second wave of data for Cabo Verde was collected in 2020. Yet, a perceived lack of safety from traffic and crime has been reported as a major driver of PA behaviors in Africa [38–40]. Thus, equitable policies to improve the population level of active transportation PA in Africa should target multilevel factors, including the provision of safe and adequate infrastructure to support walking and bicycling and the implementation of traffic control measures that reduce pedestrian exposure to high traffic volume and speed, and air and noise pollution.

Although gender, age, and education disparities in meeting WHO physical activity guidelines were relatively stable over time in most countries, we found increasing trends in gender, age, and education disparities in the prevalence of leisure-time PA across African countries. Our finding suggests African men, younger adults, and those with higher education continued to engage in more leisure-time PA over time than women, older adults, and those with less education. This is consistent with findings in other regions of the world, especially in middle-income South American countries [41]. Although disparities in PA trends have not been previously investigated in Africa, cross-sectional studies have reported persistent gender and educational disparities in low leisure-time PA among African women and less educated adults [19, 42]. Sociocultural factors, including low income and constraints associated with women’s traditional responsibilities of managing childcare and domestic chores, may make women in Africa have fewer opportunities to engage in recreational PA than men [20, 21]. High education is a good proxy for high SES [43], and compared to those less educated, the highly educated groups in Africa may be more aware of the health benefits of PA and have more financial resources to engage in recreational activities during leisure time [21, 44]. Yet, it is important to ensure equitable access to leisure-time PA and the associated health and social benefits for all populations in Africa. Future interventions to promote leisure PA should specifically target groups with higher social and economic vulnerability in Africa.

While the prevalence of transport and occupational PA increased over time in all gender and education groups, we found some trends in gender and education disparities in these domains of PA. These findings indicate that, over time, more men and those with less than high school engaged in more transport and occupational PA than women and those with higher education. This is not surprising and could reflect the widening socioeconomic disparities in the African population, driven by rapid ongoing urbanization [45, 46], globalization of unfettered capitalism [47, 48], and advances in automation and computerization of employment [49, 50]. Less educated African adults, especially women, may have low income and thus have no choice but to use active transportation modes to access many destinations and engage in manual and physically demanding occupations [20, 21]. On the other hand, more educated African adults may have more sedentary occupations and be able to own and use private cars as their transportation mode [21]. A substantial amount of transportation and occupational PA occurring in many low- and middle-income countries settings are the result of economic necessity and not due to true, free choices [51, 52]. Thus, to promote equity, it is important to improve the conditions under which necessity-driven PA occurs for a vast majority of the population [51].

Our findings have important implications for PA policy in Africa. With data from eleven African countries, and 20 countries recently reported by Strain and Colleagues [23], it appears there has been an increase in the number of African countries with surveillance data for monitoring PA trends in recent times. Yet, compared to other regions, the majority of the 54 countries in the African region still lack data on PA surveillance and monitoring, and policy [53, 54]. Only 8.3% of African countries have periodic PA surveillance systems, and about 50% do not have any PA policy plans [53]. The absence of surveillance systems in many African countries to monitor trends could undermine national and regional capacity efforts for effective planning to mitigate the rising physical inactivity-related NCDs and adverse climate and planetary health consequences in the region. Public health action to improve population-level PA and address NCDs in Africa should clearly integrate well-defined PA-promoting strategies into national PA and public health policies [55]. To be effective, such national policy efforts should leverage best practices and engage diverse stakeholders across sectors, including health and social care professionals, urban and transportation planners, architects, teachers, sport and recreation providers, educators, policymakers, and non-governmental organizations [10, 54]. Such whole-of-government and systems approaches have been recommended by the WHO and other scientific groups, including the African Physical Activity Network (AFPAN), to help countries improve population PA trends, sustainably address the burden of NCDS, and build healthier and more resilient populations [56–60].

## Strengths and limitations

The present study has some important strengths. It extends the few available African studies by examining recent secular trends in the prevalence of PA in multiple domains in 11 African countries participating in the WHO STEPS, using nationally representative data from 2003 to 2020. The WHO STEPS data source provides a comprehensive and standardized source for country-level surveillance data that could be used to monitor trends and inform policy plans. However, there are some limitations to acknowledge. Estimates of PA were based on self-reported data, which may be subject to measurement error, desirability bias, and non-precision of estimates [61, 62]. For example, meeting recommendations for sufficient levels of PA among African adults has been shown to be overestimated when based on self-report measures compared with accelerometer-derived estimates [63, 64], suggesting that the estimates reported in our study may not reflect the true population levels of PA. However, using valid and reliable self-report measures, such as the GPAQ used in the present study, represents the most feasible method for PA surveillance in Africa because of their low cost, ease of use, and ability to capture domains of PA [18, 65, 66]. Our data only included information on gender, age and education, precluding our ability to explore trends of PA by sociodemographic factors like marital status, occupation, and income that have been reported as important correlates of PA among African adults [18–21, 67]. Also, the dates for Wave 1 and Wave 2 are arbitrary, wide, and not similar between and across countries, making the interpretation of our between-country differences in PA trends challenging. However, our findings suggest a consistent pattern of within-country PA trends for most PA domains in many African countries.

## Conclusions

Overall, our data show that total, leisure-time, transportation, and occupational PA have marginally increased in most African countries since the first wave of the STEP survey (2003–2010). While women, older adults and those with lower education present disparities of lower trends in the prevalence of leisure-time PA, men and those with lower education present higher trends in transportation and occupational PA. The improvement in overall PA over time is promising, but concerted efforts and pragmatic national policy plans are needed to address the persistent prevalence of insufficient leisure-time PA and improve gender, age and education disparities in transport and occupational PA among African adults.

## Supplementary Information

Additional file 1: Supplementary Table 1. Details of each STEPS survey included in the study and country-level socioeconomic and human indices. Supplementary Table 2. Prevalence of meeting the WHO recommendations of physical activity*. Supplementary Table 3. Prevalence of some leisure-time physical activity (≥1min/week). Supplementary Table 4. Prevalence of leisure-time physical activity (≥150min/week). Supplementary Table 5. Prevalence of some transport physical activity (≥1min/week). Supplementary Table 6. Prevalence of transport physical activity (≥150min/week). Supplementary Table 7. Prevalence of some occupational physical activity (≥1min/week). Supplementary Table 8. Prevalence of occupational physical activity (≥150min/week). Supplementary Table 9. Prevalence of some leisure-time physical activity (≥1min/week) according to age groups. Supplementary Table 10. Prevalence of leisure-time physical activity (≥150min/week) according to age groups. Supplementary Table 11. Prevalence of some transport physical activity (≥1min/week) according to age groups. Supplementary Table 12. Prevalence of transport physical activity (≥150min/week) according to age groups. Supplementary Table 13. Prevalence of some occupational physical activity (≥1min/week) according to age groups. Supplementary Table 14. Prevalence of occupational physical activity (≥150min/week) according to age groups. Supplementary Table 15. Prevalence of meeting the WHO recommendations of physical activity according to age group*.

## Data Availability

The WHO STEPS Survey datasets generated and analysed during the current study are available on WHO STEPS repository, [https://extranet.who.int/ncdsmicrodata/index.php/catalog/steps/].
