# Correction to: Secular trends and sociodemographic disparities in physical activity among adults in eleven African countries: WHO STEPS 2003–2020

**DOI:** 10.1186/s12966-025-01841-5

**Published:** 2025-10-30

**Authors:** Adewale L. Oyeyemi, Raphael H.O. Araujo, Umar A. Hassan, Edward Ofori, Chad Stecher, André O. Werneck

**Affiliations:** 1 College of Health Solutions, Arizona State University, Phoenix, AZ 85004 USA; 2 Graduation Program in Health Sciences, Londrina State University, Londrina, 86057‑970 Brazil; 3 School of Public Health, The University of Queensland, Brisbane, QLD 4006 Australia; 4 Center for Epidemiological Research in Nutrition and Health, Department of Nutrition, School of Public Health, Universidade de São Paulo (USP), São Paulo, SP Brazil

**Correction to: Int J Behav Nutr Phys Act 21, 126 (2024)**

**https://doi.org/10.1186/s12966-024-01675-7**

Following the publication of the original article [1], the authors reported a typographical error in the author’s name. The incorrect name was Chad Stetcher, and it should be change to Chad Stecher.
