# Supplementary material for: Secular trends and sociodemographic disparities in physical activity among adults in eleven African countries: WHO STEPS 2003–2020
Source: Int J Behav Nutr Phys Act. 2024 Oct 30;21:126. doi: 10.1186/s12966-024-01675-7 (PMC11526601; doi:10.1186/s12966-024-01675-7)
Supplement: Supplementary file 1 — Additional file 1: Supplementary Table 1. Details of each STEPS survey included in the study and country-level socioeconomic and human indices. Supplementary Table 2. Prevalence of meeting the WHO recommendations of physical activity*. Supplementary Table 3. Prevalence of some leisure-time physical activity (≥1min/week). Supplementary Table 4. Prevalence of leisure-time physical activity (≥150min/week). Supplementary Table 5. Prevalence of some transport physical activity (≥1min/week). Supplementary Table 6. Prevalence of transport physical activity (≥150min/week). Supplementary Table 7. Prevalence of some occupational physical activity (≥1min/week). Supplementary Table 8. Prevalence of occupational physical activity (≥150min/week). Supplementary Table 9. Prevalence of some leisure-time physical activity (≥1min/week) according to age groups. Supplementary Table 10. Prevalence of leisure-time physical activity (≥150min/week) according to age groups. Supplementary Table 11. Prevalence of some transport physical activity (≥1min/week) according to age groups. Supplementary Table 12. Prevalence of transport physical activity (≥150min/week) according to age groups. Supplementary Table 13. Prevalence of some occupational physical activity (≥1min/week) according to age groups. Supplementary Table 14. Prevalence of occupational physical activity (≥150min/week) according to age groups. Supplementary Table 15. Prevalence of meeting the WHO recommendations of physical activity according to age group*. [file 12966_2024_1675_MOESM1_ESM.docx]

**Supplementary Table 1**. Details of each STEPS survey included in the study and country-level socioeconomic and human indices

| **Country (year)** | **Sample** | **Age range** | **Coverage** | **Country income classification** | **Country Human Development Index** |
| --- | --- | --- | --- | --- | --- |
| Algeria (2003) | 4,097  279 missings | 25-64 | Setif and Mostaganem | Lower-middle income | 0.276 |
| Algeria (2016) | 6,989  544 missings | 18-64 | National | Upper-middle income | 0.372 |
| Benin (2008) | 6,842  674 missings | 25-64 | National | Low-income | 0.470 |
| Benin (2015) | 5,126  325 missings | 18-69 | National | Low-income | 0.509 |
| Botswana (2007) | 4,003  896 missings | 25-64 | National | Upper-middle income | 0.629 |
| Botswana (2014) | 3,902  496 missings | 18-69 | National | Upper-middle income | 0.683 |
| Cabo Verde (2007) | 1,760  138 missings | 25-64 | National | Lower-middle income | 0.639 |
| Cabo Verde (2020) | 4,563  524 missings | 18-69 | National | Lower-middle income | 0.649 |
| Eritrea (2004) | 2,183  758 missings | 18-64 | National | Low-income | 0.466 |
| Eritrea (2010) | 6,265  304 missings | 25-74 | National | Low-income | 0.458 |
| Eswatini (2007) | 1,288  67 missings | 25-64 | National | Lower-middle income | 0.464 |
| Eswatini (2014) | 3,259  597 missings | 15-69 | National | Lower middle income | 0.560 |
| Malawi (2009) | 3,259  1,414 missings | 25-64 | National | Low-income | 0.452 |
| Malawi (2017) | 4,187  1,144 missings | 18-69 | National | Low-income | 0.506 |
| Mali (2007) | 2,401  1,011 missings | 18-64 | District of Bamako, Commune of Kati central and Commune of Ouéléssebougou | Low-income | 0.381 |
| Mali (2013) | 1,950  721 missings | 18-65 | Kati, Ouéléssébougou, Koulikoro, Ségou and Bamako District | Low-income | 0.404 |
| Central African Republic (2010) | 4,029  711 missings | 25-64 | Bangui | Low-income | 0.357 |
| Central African Republic (2017) | 3,301  430 missings | 15-64 | Bangui and Ombella M'Poko | Low-income | 0.379 |
| Sao Tome and Principe (2009) | 2,457  310 missings | 25-64 | National | Lower-middle income | 0.550 |
| Sao Tome and Principe (2019) | 2,418  167 missings | 18-69 | National | Lower-middle income | 0.607 |
| Zambia (2008) | 1,912  638 missings | 25-90 | Lusaka district | Low-income | 0.506 |
| Zambia (2017) | 4,302  863 missings | 25-69 | National | Lower-middle income | 0.568 |

Note. OPA, occupational physical activity. Note: country income level is based on the World Bank income classification of countries into high-income country, upper-middle income country, lower-middle income country, and low-income country for the years the surveys were conducted in each country [1]. Human Development Index (HDI) was based on the United Nations Development Programme (UNDP) HDI data calculated from life expectancy at birth, mean and expected years of schooling, and gross national income per capital for the years the surveys were conducted in each country [2]; greater scores represent greater human development.

[1] https://ourworldindata.org/grapher/world-bank-income-groups?time=2008&region=Africa

[2] https://hdr.undp.org/data-center/human-development-index#/indicies/HDI

**Supplementary Table 2**. Prevalence of meeting the WHO recommendations of physical activity*.

|  | Gender | |  |  | Education | | | |
| --- | --- | --- | --- | --- | --- | --- | --- | --- |
|  | Women  % (95%CI) | Men  % (95%CI) | gap  p.p. |  | No education  % (95%CI) | Less than high school  % (95%CI) | High school or more  % (95%CI) | gap  p.p. |
| *Country (year)* |  |  |  |  |  |  |  |  |
| Algeria (2003) | 60.1 (58.1 - 62.1) | 72.9 (70.6 - 75.1) | -12.8 |  | 58.6 (56.4 - 60.9) | 69.0 (66.5 - 71.3) | 75.5 (71.8 - 78.8) | -16.9 |
| Algeria (2016) | 66.0 (64.4 - 67.5) | 85.9 (84.6 - 87.2) | -19.9 |  | 64.0 (60.9 - 66.9) | 77.7 (76.0 - 79.3) | 77.5 (75.9 - 79.0) | -13.5 |
| Benin (2008) | 92.1 (91.1 - 93.0) | 95.1 (94.3 - 95.8) | -3.0 |  | 94.7 (93.9 - 95.3) | 93.0 (91.7 - 94.2) | 89.2 (86.7 - 91.2) | 5.5 |
| Benin (2015) | 80.6 (78.6 - 82.5) | 85.8 (83.8 - 87.6) | -5.2 |  | 82.1 (80.0 - 84.0) | 83.0 (80.6 - 85.2) | 85.7 (82.5 - 88.5) | -3.6 |
| Botswana (2007) | 68.9 (66.6 - 71.1) | 78.5 (75.0 - 81.7) | -9.6 |  | 63.4 (56.6 - 69.7) | 73.6 (70.5 - 76.4) | 76.6 (74.2 - 78.9) | -13.2 |
| Botswana (2014) | 72.2 (60.7 - 75.6) | 84.6 (81.6 - 87.2) | -12.4 |  | 79.4 (74.7 - 83.3) | 80.1 (77.1 - 82.9) | 76.3 (73.1 - 79.2) | 2.7 |
| Cabo Verde (2007) | 72.7 (69.0 - 76.1) | 87.9 (84.4 - 90.7) | -15.2 |  | 74.8 (67.4 - 81.0) | 83.2 (79.9 - 86.0) | 77.0 (72.0 - 81.4) | -2.2 |
| Cabo Verde (2020) | 54.3 (51.6 - 57.0) | 75.5 (72.5 - 78.3) | -21.2 |  | 54.1 (46.0 - 62.0) | 69.8 (67.0 - 72.5) | 62.5 (59.6 - 65.3) | -8.4 |
| Eritrea (2004) | 54.5 (51.0 - 57.9) | 77.4 (73.9 - 80.5) | -22.9 |  | 59.4 (55.1 - 63.6) | 64.3 (59.5 - 68.7) | 70.0 (65.8 - 73.8) | -10.5 |
| Eritrea (2010) | 86.0 (84.8 - 87.2) | 93.5 (92.1 - 94.6) | -7.5 |  | 85.5 (84.1 - 86.9) | 90.0 (88.5 - 91.4) | 87.1 (83.4 - 90.1) | -1.6 |
| Eswatini (2007) | 66.8 (63.5 - 70.1) | 73.7 (69.1 - 77.8) | -6.9 |  | 69.7 (63.5 - 75.2) | 71.3 (67.1 - 75.1) | 68.4 (63.9 - 72.7) | 1.3 |
| Eswatini (2014) | 76.6 (74.0 - 79.1) | 88.0 (85.1 - 90.5) | -11.4 |  | 85.2 (80.0 - 89.3) | 82.9 (79.9 - 85.6) | 80.2 (77.3 - 82.8) | 4.8 |
| Malawi (2009) | 92.6 (91.5 - 93.5) | 95.9 (94.6 - 96.9) | -3.3 |  | 93.9 (92.0 - 95.3) | 94.9 (93.9 - 95.7) | 92.0 (89.4 - 94.0) | 1.9 |
| Malawi (2017) | 97.9 (97.0 - 98.5) | 98.9 (97.8 - 99.4) | -1.0 |  | 97.2 (94.5 - 98.6) | 98.8 (98.0 - 99.3) | 97.8 (96.3 - 98.7) | -0.6 |
| Mali (2007) | 37.9 (34.7 - 41.2) | 51.6 (47.4 - 55.8) | -13.7 |  | 41.7 (37.2 - 46.3) | 43.6 (39.5 - 47.6) | 44.6 (39.6 - 49.7) | -3.1 |
| Mali (2013) | 65.7 (62.2 - 69.0) | 64.6 (60.2 - 68.7) | 1.1 |  | 64.0 (59.7 - 68.1) | 69.1 (64.5 - 73.4) | 62.0 (56.4 - 67.3) | 2.4 |
| Central African Republic (2010) | 79.8 (76.9 - 82.5) | 85.0 (82.5 - 87.2) | -5.2 |  | 72.1 (66.1 - 77.4) | 83.9 (81.3 - 86.2) | 85.6 (82.7 - 88.0) | -13.5 |
| Central African Republic (2017) | 93.7 (92.4 - 94.8) | 96.1 (94.8 - 97.0) | -2.4 |  | 88.6 (85.1 - 91.4) | 95.0 (93.6 - 96.1) | 96.2 (94.9 - 97.1) | -7.6 |
| Sao Tome and Principe (2008) | 81.6 (79.4 - 83.7) | 91.9 (89.8 - 93.5) | -10.3 |  | 77.5 (71.5 - 82.5) | 87.8 (85.6 - 89.6) | 86.6 (84.2 - 88.7) | -9.1 |
| Sao Tome and Principe (2019) | 79.7 (77.1 - 82.0) | 86.0 (83.0 - 88.5) | -6.3 |  | 76.1 (66.9 - 83.4) | 81.3 (79.0 - 83.5) | 83.9 (79.2 - 87.6) | -7.8 |
| Zambia (2008) | 84.7 (82.3 - 86.9) | 81.6 (77.0 - 85.4) | 3.1 |  | 68.8 (57.7 - 78.2) | 86.7 (84.0 - 89.0) | 82.2 (78.5 - 85.4) | -13.4 |
| Zambia (2017) | 84.7 (82.7 - 86.5) | 93.9 (92.3 - 95.3) | -9.2 |  | 87.6 (83.5 - 90.7) | 91.4 (89.9 - 92.6) | 84.1 (80.8 - 86.9) | 3.5 |
| **Total wave 1** | 72.0 (64.3 - 79.7) | 81.3 (76.8 - 85.8) | -9.3 |  | 70.5 (61.9 - 79.2) | 77.6 (72.1 - 83.2) | 77.3 (72.1 - 82.5) | -6.8 |
| **Total wave 2** | 78.0 (67.8 - 88.2) | 86.8 (82.3 - 91.2) | -8.8 |  | 78.5 (69.4 - 87.7) | 83.6 (77.1 - 90.1) | 81.3 (73.9 - 88.7) | -2.8 |

Note. *Using WHO 2020 guidelines on physical activity of at least 75min/week of vigorous physical activity or 150min/week of the combination of moderate and vigorous physical activity.

**Supplementary Table 3**. Prevalence of some leisure-time physical activity (≥1min/week).

|  | Gender | |  |  | Education | | | |
| --- | --- | --- | --- | --- | --- | --- | --- | --- |
|  | Women  % (95%CI) | Men  % (95%CI) | gap  p.p. |  | No education  % (95%CI) | Less than high school  % (95%CI) | High school or more  % (95%CI) | gap  p.p. |
| *Country (year)* |  |  |  |  |  |  |  |  |
| Algeria (2003) | 0.4 (0.2 - 0.7) | 2.2 (1.6 - 3.1) | -1.8 |  | 0.2 (0.1 - 0.6) | 1.2 (0.7 - 1.9) | 3.5 (2.3 - 5.4) | -3.3 |
| Algeria (2016) | 5.9 (5.1 - 6.8) | 24.4 (22.7 - 26.3) | -18.5 |  | 1.3 (0.8 - 2.3) | 12.6 (11.2 - 14.2) | 20.7 (19.0 - 22.4) | -19.4 |
| Benin (2008) | 4.1 (3.5 - 4.9) | 12.6 (11.5 - 13.8) | -8.5 |  | 4.4 (3.8 - 5.1) | 10.3 (8.9 - 11.9) | 25.4 (22.4 - 28.7) | -21.0 |
| Benin (2015) | 3.9 (3.0 - 5.2) | 17.2 (14.9 - 19.7) | -13.3 |  | 2.9 (2.1 - 4.0) | 10.9 (8.7 - 13.6) | 26.4 (22.3 - 30.8) | -23.5 |
| Botswana (2007) | 6.0 (5.0 - 7.2) | 22.7 (19.9 - 25.8) | -16.7 |  | 4.3 (2.3 - 7.9) | 6.9 (5.2 - 8.9) | 23.0 (20.6 - 25.7) | -18.7 |
| Botswana (2014) | 14.1 (12.1 - 16.5) | 38.3 (34.3 - 42.6) | -24.2 |  | 11.9 (7.7 - 17.7) | 24.6 (21.1 - 28.5) | 32.3 (28.5 - 36.3) | -20.4 |
| Cabo Verde (2007) | 4.7 (3.0 - 7.2) | 33.2 (27.5 - 39.5) | -28.5 |  | 4.8 (2.1 - 10.5) | 10.9 (7.8 - 15) | 33.3 (26.6 - 40.8) | -28.5 |
| Cabo Verde (2020) | 13.8 (12 - 15.7) | 43.6 (40.3 - 47) | -29.8 |  | 4.8 (2.5 - 9.1) | 17.4 (15.1 - 20) | 37.1 (34.2 - 40.1) | -32.3 |
| Eritrea (2004) | 0.8 (0.3 - 1.7) | 5.9 (4.3 - 8.0) | -5.1 |  | 0.8 (0.3 - 2.1) | 2.4 (1.3 - 4.4) | 5.8 (4.0 – 8.2) | -5.0 |
| Eritrea (2010) | 0.9 (0.6 - 1.2) | 4.0 (3.1 - 5.1) | -3.1 |  | 0.5 (0.3 - 0.9) | 1.3 (0.9 - 1.9) | 5.4 (3.9 - 7.4) | -4.9 |
| Eswatini (2007) | 13.1 (10.9 - 15.6) | 25.5 (21.3 - 30.2) | -12.4 |  | 13.9 (9.9 - 19.1) | 15.9 (12.7 - 19.7) | 23.2 (19.3 - 27.7) | -9.3 |
| Eswatini (2014) | 15.1 (12.6 – 18.0) | 46.1 (41.7 - 50.6) | -31.0 |  | 8.2 (4.9 - 13.4) | 22.8 (19.6 - 26.3) | 36.6 (32.7 - 40.6) | -28.4 |
| Malawi (2009) | 15.4 (14 - 16.8) | 30.6 (28.0 - 33.3) | -15.2 |  | 9.6 (7.7 - 12) | 22.0 (20.2 - 23.9) | 42.4 (38 - 46.8) | -32.8 |
| Malawi (2017) | 24.5 (21.2 - 28) | 45.5 (38.7 - 52.4) | -21.0 |  | 8.7 (5.5 - 13.5) | 32.0 (27.9 - 36.4) | 50.8 (41.6 - 59.9) | -42.1 |
| Mali (2007) | 1.7 (1.0 - 2.8) | 7.4 (5.4 - 9.9) | -5.7 |  | 1.1 (0.5 - 2.7) | 3.5 (2.3 - 5.3) | 7.8 (5.5 - 11.0) | -6.7 |
| Mali (2013) | 0.0 (0.0 - 0.0) | 0.8 (0.3 – 2.2) | -0.8 |  | 0.2 (0.0 - 1.4) | 0.7 (0.2 - 2.2) | 0.0 (0.0 - 0.0) | 0.2 |
| Central African Republic (2010) | 5.6 (4.2 - 7.3) | 28.4 (25.4 - 31.7) | -22.8 |  | 3.6 (1.9 - 6.8) | 13.6 (11.1 - 16.4) | 26.6 (23.4 - 30.0) | -23.0 |
| Central African Republic (2017) | 9.5 (8.0 - 11.2) | 39.1 (35.8 - 42.5) | -29.6 |  | 6.6 (4.0 - 10.7) | 13.1 (10.9 - 15.8) | 37.4 (34.2 - 40.7) | -30.8 |
| Sao Tome and Principe (2008) | 9.1 (7.7 - 10.8) | 30.8 (27.9 - 34.0) | -21.7 |  | 10.4 (7.0 - 15) | 14.2 (12.1 - 16.5) | 26.4 (23.6 - 29.5) | -16.0 |
| Sao Tome and Principe (2019) | 11.1 (9.2 - 13.2) | 40.0 (36.2 - 43.8) | -28.9 |  | 3.2 (1.0 - 9.7) | 17.2 (15.2 - 19.4) | 33.0 (28.4 - 38.0) | -29.8 |
| Zambia (2008) | 19.2 (16.8 - 21.8) | 37.7 (32.6 - 43.1) | -18.5 |  | 6.5 (2.7 - 14.7) | 20.4 (17.6 - 23.6) | 31.9 (27.9 - 36.2) | -25.4 |
| Zambia (2017) | 11.7 (10.1 - 13.4) | 30.0 (27.0 - 33.3) | -18.3 |  | 6 (3.1 - 11.1) | 18.7 (16.7 - 20.9) | 29.1 (25.1 - 33.4) | -23.1 |
| **Total wave 1** | 7.2 (2.7-11.8) | 21.4 (16.9-25.9) | -14.2 |  | 5.2 (2.1-8.3) | 11.0 (5.7 - 16.2) | 22.7 (15.8 - 29.5) | -17.5 |
| **Total wave 2** | 11.1 (7.3-14.8) | 29.9 (22.8-37.1) | -18.8 |  | 4.9 (3.8-6.0) | 15.6 (9.5 - 21.6) | 30.9 (22.5 - 39.2) | -26.0 |

**Supplementary Table 4**. Prevalence of leisure-time physical activity (≥150min/week).

|  | Gender | |  |  | Education | | | |
| --- | --- | --- | --- | --- | --- | --- | --- | --- |
|  | Women  % (95%CI) | Men  % (95%CI) | gap  p.p. |  | No education  % (95%CI) | Less than high school  % (95%CI) | High school or more  % (95%CI) | gap  p.p. |
| *Country (year)* |  |  |  |  |  |  |  |  |
| Algeria (2003) | 0.3 (0.1 - 0.6) | 1.7 (1.1 - 2.5) | -1.4 |  | 0.2 (0.1 - 0.6) | 0.9 (0.5 - 1.6) | 2.5 (1.5 - 4.1) | -2.3 |
| Algeria (2016) | 3.7 (3.1 - 4.5) | 16.7 (15.2 - 18.4) | -13 |  | 1.0 (0.5 - 1.8) | 8.1 (6.9 - 9.4) | 14.3 (12.9 - 15.9) | -13.3 |
| Benin (2008) | 3.7 (3.1 - 4.4) | 8.4 (7.5 - 9.4) | -4.7 |  | 3.7 (3.2 - 4.4) | 6.7 (5.6 - 8.0) | 17.0 (14.4 - 19.9) | -13.3 |
| Benin (2015) | 2.5 (1.8 - 3.5) | 11.5 (9.7 - 13.7) | -9.0 |  | 2.3 (1.6 - 3.4) | 6.8 (5.1 - 9.1) | 17.2 (13.9 - 21.1) | -14.9 |
| Botswana (2007) | 4.5 (3.7 - 5.5) | 17.6 (15.1 - 20.4) | -13.1 |  | 3.7 (1.9 - 7.3) | 4.6 (3.3 - 6.5) | 18.2 (16.0 - 20.6) | -14.5 |
| Botswana (2014) | 10.5 (8.7 - 12.6) | 30.1 (26.3 - 34.3) | -19.6 |  | 9.4 (5.6 - 15.3) | 19.5 (16.2 - 23.2) | 24.4 (21.0 - 28.2) | -15.0 |
| Cabo Verde (2007) | 3.9 (2.4 - 6.4) | 26.7 (21.2 - 33.2) | -22.8 |  | 4.8 (2.1 - 10.5) | 7.0 (4.5 - 10.8) | 29.4 (22.7 - 37.0) | -24.6 |
| Cabo Verde (2020) | 11.4 (9.7 - 13.2) | 35.4 (32.2 - 38.7) | -24.0 |  | 4.2 (2.0 - 8.7) | 12.9 (10.9 - 15.2) | 31.1 (28.3 - 34.0) | -26.9 |
| Eritrea (2004) | 0.8 (0.3 - 1.7) | 4.0 (2.7 - 5.8) | -3.2 |  | 0.8 (0.3 - 2.1) | 1.7 (0.8 - 3.5) | 4.0 (2.6 - 6.1) | -3.2 |
| Eritrea (2010) | 0.7 (0.5 - 1.0) | 3.5 (2.7 - 4.6) | -2.8 |  | 0.4 (0.2 - 0.7) | 1.2 (0.8 - 1.7) | 4.7 (3.3 - 6.7) | -4.3 |
| Eswatini (2007) | 10.9 (8.9 - 13.3) | 20.3 (16.5 - 24.7) | -9.4 |  | 11 (7.4 - 16.1) | 12.7 (9.9 - 16.3) | 19.1 (15.5 - 23.3) | -8.1 |
| Eswatini (2014) | 12.1 (9.7 - 14.9) | 38.1 (33.9 - 42.6) | -26.0 |  | 5.8 (3.3 – 10.0) | 18.5 (15.6 - 21.9) | 30.2 (26.4 - 34.2) | -24.4 |
| Malawi (2009) | 9.7 (8.6 - 10.9) | 21.5 (19.3 - 23.9) | -11.8 |  | 6.7 (5.1 - 8.8) | 15.0 (13.5 - 16.7) | 28 (24.1 - 32.3) | -21.3 |
| Malawi (2017) | 17.2 (14.3 - 20.6) | 33.4 (26.6 - 41) | -16.2 |  | 5.8 (3.1 - 10.5) | 21.5 (18.1 - 25.4) | 40.8 (30.9 - 51.6) | -35.0 |
| Mali (2007) | 1.3 (0.7 - 2.3) | 7.0 (5.1 - 9.5) | -5.7 |  | 0.9 (0.3 - 2.4) | 3.1 (2.0 - 4.9) | 7.3 (5.0 - 10.4) | -6.4 |
| Mali (2013) | 0.0 (0.0 – 0.0) | 0.8 (0.3 – 2.2) | -0.8 |  | 0.2 (0.0 - 1.4) | 0.7 (0.2 - 2.2) | 0.0 (0.0 - 0.0) | 0.2 |
| Central African Republic (2010) | 4.2 (3.1 - 5.8) | 19.6 (17 - 22.5) | -15.4 |  | 2.6 (1.2 - 5.7) | 9.2 (7.3 - 11.6) | 18.9 (16.2 - 22.0) | -16.3 |
| Central African Republic (2017) | 7.1 (5.8 - 8.6) | 26.2 (23.2 - 29.3) | -19.1 |  | 4.7 (2.7 - 8.3) | 8.4 (6.6 - 10.6) | 26.0 (23.1 - 29.1) | -21.3 |
| Sao Tome and Principe (2008) | 6.8 (5.6 - 8.4) | 20.4 (17.9 - 23.2) | -13.6 |  | 7 (4.3 - 11.1) | 10.0 (8.3 - 12) | 17.9 (15.4 - 20.6) | -10.9 |
| Sao Tome and Principe (2019) | 7.5 (6.1 - 9.2) | 24.5 (21.3 - 27.9) | -17.0 |  | 2.2 (0.5 - 9.8) | 10.5 (9 - 12.3) | 22.7 (18.8 - 27.1) | -20.5 |
| Zambia (2008) | 14.8 (12.6 - 17.2) | 27.0 (22.5 - 32.1) | -12.2 |  | 3.9 (1.3 - 11.4) | 16.0 (13.5 - 18.8) | 23.0 (19.4 - 26.9) | -19.1 |
| Zambia (2017) | 8.2 (7.0 - 9.8) | 23.2 (20.3 - 26.3) | -15.0 |  | 3.4 (1.5 - 7.5) | 14.4 (12.5 - 16.5) | 21.4 (17.9 - 25.4) | -18.0 |
| **Total wave 1** | 5.5 (2.8 - 8.1) | 15.7 (12.2 - 19.1) | -10.2 |  | 3.9 (1.8 - 6.0) | 7.8 (4.2 - 11.5) | 16.8 (12.8 - 20.9) | -12.9 |
| **Total wave 2** | 8.1 (5.5 - 10.7) | 22.1 (16.8 - 27.5) | -14.0 |  | 3.5 (2.8 - 4.3) | 11.1 (7.1 - 15.2) | 23.3 (16.5 - 30.1) | -19.8 |

**Supplementary Table 5**. Prevalence of some transport physical activity (≥1min/week).

|  | Gender | |  |  | Education | | | |
| --- | --- | --- | --- | --- | --- | --- | --- | --- |
|  | Women%  (95%CI) | Men  % (95%CI) | gap  p.p. |  | No education  % (95%CI) | Less than high school  % (95%CI) | High school or more  % (95%CI) | gap  p.p. |
| *Country (year)* |  |  |  |  |  |  |  |  |
| Algeria (2003) | 32.2 (30.4 - 34.2) | 71.5 (69.1 - 73.7) | -39.3 |  | 36.3 (34.1 - 38.5) | 54.0 (51.4 - 56.6) | 66.4 (62.5 - 70.1) | -30.1 |
| Algeria (2016) | 66.5 (64.9 - 68.1) | 85.8 (84.4 - 87.1) | -19.3 |  | 65.1 (62.1 - 68.1) | 76.4 (74.7 - 78.0) | 78.6 (77.1 - 80.1) | -13.5 |
| Benin (2008) | 93.6 (92.6 - 94.4) | 93.8 (92.9 - 94.6) | -0.2 |  | 94.5 (93.8 - 95.2) | 92.2 (90.8 - 93.4) | 92.1 (89.9 - 93.8) | 2.4 |
| Benin (2015) | 80.2 (78.2 - 82.1) | 81.1 (79.0 - 83.0) | -0.9 |  | 81.3 (79.2 - 83.2) | 78.5 (75.9 - 81.0) | 82.2 (78.9 - 85.1) | -0.9 |
| Botswana (2007) | 79.8 (77.8 - 81.6) | 80.2 (76.9 - 83.2) | -0.4 |  | 72.9 (66.4 - 78.6) | 81.6 (78.8 - 84.1) | 81.2 (78.8 - 83.4) | -8.3 |
| Botswana (2014) | 76.2 (73.6 - 78.6) | 81.2 (77.7 - 84.2) | -5.0 |  | 79.3 (73.6 – 84.1) | 82.7 (79.8 - 82.7) | 74.4 (70.7 - 77.7) | 4.9 |
| Cabo Verde (2007) | 84.1 (81.1 - 86.8) | 87.5 (83.9 - 90.4) | -3.4 |  | 82.0 (74.6 - 87.7) | 89.8 (87.1 - 92.0) | 81.1 (76.4 - 85.1) | 0.9 |
| Cabo Verde (2020) | 67.3 (64.8 - 69.8) | 69.1 (65.9 - 72.1) | -1.8 |  | 64.3 (56.1 - 71.8) | 72.0 (69.3 - 74.6) | 66.1 (63.2 - 68.9) | -1.8 |
| Eritrea (2004) | 75.6 (72.6 - 78.5) | 87.1 (84.2 - 89.5) | -11.5 |  | 76.8 (72.9 - 80.3) | 80.1 (76.0 - 83.7) | 85.1 (81.7 - 87.9) | -8.3 |
| Eritrea (2010) | 76.8 (75.3 - 78.2) | 83.2 (81.1 - 85.0) | -6.4 |  | 75.1 (73.3 - 76.8) | 80.1 (78.1 - 82.1) | 83.4 (79.4 - 86.7) | -8.3 |
| Eswatini (2007) | 64.7 (61.3 - 68.0) | 64.0 (59.1 - 68.6) | 0.7 |  | 60.9 (54.4 - 67.1) | 66.8 (62.5 - 70.9) | 63.3 (58.6 - 67.8) | -2.4 |
| Eswatini (2014) | 70.4 (67.5 - 73.1) | 78.1 (74.0 - 81.6) | -7.7 |  | 69.2 (62.3 - 75.3) | 73.0 (69.1 - 76.6) | 75.1 (71.8 – 78.1) | -5.9 |
| Malawi (2009) | 78.9 (77.3 - 80.5) | 88.0 (86.0 - 89.7) | -9.1 |  | 81.0 (78.2 - 83.4) | 83.6 (82.0 - 85.1) | 85.2 (81.8 - 88.0) | -4.2 |
| Malawi (2017) | 88.1 (85.6 - 90.2) | 93.1 (90.3 - 95.1) | -5.0 |  | 83.1 (77.4 - 87.6) | 92.0 (89.9 - 93.7) | 89.7 (85.1 - 93.0) | -6.6 |
| Mali (2007) | 55.3 (51.9 - 58.6) | 64.6 (60.5 - 68.6) | -9.3 |  | 56.3 (51.6 - 60.9) | 59.6 (55.5 - 63.5) | 61.0 (60.0 - 63.5) | -4.7 |
| Mali (2013) | 76.1 (72.9 – 79.1) | 75.2 (71.3 - 78.8) | 0.9 |  | 73.4 (69.3 – 77.1) | 77.2 (72.9 - 81.0) | 76.0 (70.9 - 80.4) | -2.6 |
| Central African Republic (2010) | 83.7 (81.0 - 86.2) | 87.4 (85.0 - 89.4) | -3.7 |  | 77.2 (71.3 - 82.2) | 86.4 (84.0 - 88.6) | 88.5 (85.9 - 90.7) | -11.3 |
| Central African Republic (2017) | 92.0 (90.6 - 93.3) | 92.5 (90.6 - 94.0) | -0.5 |  | 87.9 (84.0 - 90.9) | 93.0 (91.2 - 94.4) | 92.6 (90.8 - 94.1) | -4.7 |
| Sao Tome and Principe (2008) | 73.2 (70.7 - 75.6) | 80.1 (77.3 - 82.7) | -6.9 |  | 73.4 (67.3 - 78.8) | 79.0 (76.5 - 81.4) | 73.8 (70.8 - 76.6) | -0.4 |
| Sao Tome and Principe (2019) | 57.3 (54.2 - 60.3) | 61.1 (57.2 - 64.9) | -3.8 |  | 47.2 (37.6 - 57.0) | 59.5 (56.6 - 62.2) | 57.9 (52.4 - 63.2) | -10.7 |
| Zambia (2008) | 91.9 (90.0 - 93.5) | 93.3 (90.0 - 95.5) | -1.4 |  | 81.8 (71.6 - 88.9) | 94.4 (92.5 - 95.9) | 90.7 (87.7 - 93.0) | -8.9 |
| Zambia (2017) | 81.0 (78.8 - 83.1) | 89.6 (87.4 - 91.3) | -8.6 |  | 77.7 (71.9 - 82.6) | 87.2 (85.5 - 88.8) | 81.9 (78.3 - 85.1) | -4.2 |
| **Total wave 1** | 74.2 (71.1 - 77.4) | 82.8 (81.2 - 84.5) | -8.6 |  | 72.2 (67.7 - 76.7) | 79.6 (77.1 - 82.1) | 80.3 (78.2 - 82.3) | -8.1 |
| **Total wave 2** | 75.6 (69.2 - 82.0) | 81.0 (78.2 - 83.8) | -5.4 |  | 73.0 (67.7 - 78.3) | 79.2 (74.3 - 84.2) | 78.0 (74.4 - 81.6) | -5.0 |

**Supplementary Table 6**. Prevalence of transport physical activity (≥150min/week).

|  | Gender | |  |  | Education | | | |
| --- | --- | --- | --- | --- | --- | --- | --- | --- |
|  | Women  % (95%CI) | Men  % (95%CI) | gap  p.p. |  | No education  % (95%CI) | Less than high school  % (95%CI) | High school or more  % (95%CI) | gap  p.p. |
| *Country (year)* |  |  |  |  |  |  |  |  |
| Algeria (2003) | 18.7 (17.1 - 20.3) | 55.1 (52.6 - 57.6) | -36.4 |  | 24.3 (22.4 - 26.4) | 38.1 (35.6 - 40.7) | 46.4 (42.4 - 50.4) | -22.1 |
| Algeria (2016) | 38.0 (36.4 - 39.7) | 68.9 (67.1 - 70.7) | -30.9 |  | 41.7 (38.6 - 44.8) | 53.8 (51.8 - 55.8) | 56.0 (54.1 - 57.8) | -14.3 |
| Benin (2008) | 81.9 (80.5 - 83.2) | 85.4 (84.1 - 86.6) | -3.5 |  | 85.8 (84.6 - 86.8) | 82.1 (80.1 - 83.9) | 75.9 (72.7 - 78.9) | 9.9 |
| Benin (2015) | 59.2 (56.7 - 61.6) | 61.5 (58.8 - 64.2) | -2.3 |  | 61.6 (59.1 - 64.1) | 59.8 (56.5 - 62.9) | 58.3 (53.8 - 62.7) | 3.3 |
| Botswana (2007) | 39.5 (37.1 - 41.8) | 51.0 (47.2 - 54.7) | -11.5 |  | 42.4 (36.0 - 49.1) | 46.4 (43.1 - 49.8) | 43.9 (41.0 - 46.9) | -1.5 |
| Botswana (2014) | 43.8 (41.0 - 46.7) | 58.7 (54.6 - 62.8) | -14.9 |  | 59.2 (53.1 - 65.0) | 56.2 (52.4 - 59.9) | 43.7 (39.8 - 47.8) | 15.5 |
| Cabo Verde (2007) | 52.0 (47.7 - 56.3) | 55.6 (49.6 - 61.5) | -3.6 |  | 61.9 (54.3 - 69.0) | 62.0 (57.3 - 66.4) | 39.5 (33.1 - 46.3) | 22.4 |
| Cabo Verde (2020) | 32.4 (29.9 - 35.0) | 37.8 (34.7 - 41.1) | -5.4 |  | 37.5 (30.1 - 45.5) | 42.1 (39.0 - 45.3) | 30.6 (27.9 - 33.4) | 6.9 |
| Eritrea (2004) | 47.6 (44.1 - 51.0) | 71.8 (68.2 - 75.2) | -24.2 |  | 54.1 (49.7 - 58.4) | 57.3 (52.5 - 62.0) | 63.2 (58.9 - 67.3) | -9.1 |
| Eritrea (2010) | 59.5 (57.8 - 61.2) | 74.3 (72.0 - 76.6) | -14.8 |  | 60.2 (58.1 - 62.2) | 65.2 (62.7 - 67.6) | 61.4 (56.7 - 65.9) | -1.2 |
| Eswatini (2007) | 39.9 (36.5 - 43.4) | 43.4 (38.6 - 48.4) | -3.5 |  | 43.0 (36.6 - 49.6) | 44.8 (40.3 - 49.3) | 37.4 (32.9 - 42.1) | 5.6 |
| Eswatini (2014) | 36.0 (32.9 - 39.2) | 52.4 (47.9 - 56.8) | -16.4 |  | 43.2 (36.4 - 50.3) | 43.9 (39.9 - 48.0) | 42.7 (38.9 - 46.6) | 0.5 |
| Malawi (2009) | 48.4 (46.4 - 50.3) | 61.3 (58.5 - 64.0) | -12.9 |  | 49.4 (46.0 - 52.9) | 56.2 (54.1 - 58.3) | 55.4 (51.0 - 59.8) | -6.0 |
| Malawi (2017) | 64.9 (61.3 - 68.4) | 79.2 (74.6 - 83.1) | -14.3 |  | 63.4 (56.6 - 69.6) | 74.4 (70.9 - 77.7) | 68.8 (61.1 - 75.6) | -5.4 |
| Mali (2007) | 32.7 (29.6 - 35.9) | 43.3 (39.2 - 47.5) | -10.6 |  | 36.9 (32.6 - 41.5) | 36.9 (33.1 - 41.0) | 36.6 (31.8 – 41.6) | 0.3 |
| Mali (2013) | 62.1 (58.5 - 65.5) | 60.0 (55.6 - 64.3) | 2.1 |  | 60.4 (56.0 - 64.6) | 64.8 (60.2 – 69.3) | 57.8 (52.2 - 63.2) | 2.6 |
| Central African Republic (2010) | 72.6 (69.6 - 75.5) | 78.8 (75.9 - 81.3) | -6.2 |  | 66.3 (60.3 - 71.8) | 77.1 (74.1 - 79.8) | 78.5 (75.4 - 81.4) | -12.2 |
| Central African Republic (2017) | 86.0 (84.2 - 87.7) | 87.9 (85.6 - 89.8) | -1.9 |  | 80.7 (76.3 - 84.5) | 87.6 (85.4 - 89.5) | 87.7 (85.5 - 89.6) | -7.0 |
| Sao Tome and Principe (2008) | 44.0 (41.3 - 46.7) | 57.7 (54.4 - 61.0) | -13.7 |  | 43.3 (36.9 - 49.9) | 54.3 (51.2 - 57.3) | 47.0 (43.7 - 50.3) | -3.7 |
| Sao Tome and Principe (2019) | 26.1 (23.6 - 28.8) | 36.9 (33.3 - 40.6) | -10.8 |  | 21.8 (15.1 - 30.3) | 29.6 (27.1 - 32.2) | 30.6 (26.1 - 35.5) | -8.8 |
| Zambia (2008) | 42.3 (39.2 - 45.5) | 58.6 (53.2 - 63.8) | -16.3 |  | 39.0 (28.8 - 50.2) | 49.4 (45.8 - 53.1) | 43.3 (38.9 - 47.7) | -4.3 |
| Zambia (2017) | 50.0 (47.3 - 52.6) | 67.4 (64.1 - 70.5) | -17.4 |  | 51.0 (44.2 - 57.8) | 62.4 (59.9 - 64.8) | 50.4 (45.9 - 55.0) | 0.6 |
| **Total wave 1** | 47.3 (40.2 - 54.4) | 60.3 (54.6 - 66.1) | -13.0 |  | 49.9 (41.8 - 58.0) | 55.1 (49.1 - 61.1) | 51.6 (43.4 - 59.9) | -1.7 |
| **Total wave 2** | 50.7 (42.7 - 58.7) | 62.3 (57.8 - 66.7) | -11.6 |  | 52.7 (45.7 - 59.6) | 58.1 (51.7 - 64.5) | 53.4 (48.2 - 58.6) | -0.7 |

**Supplementary Table 7**. Prevalence of some occupational physical activity (≥1min/week).

|  | Gender | |  |  | Education | | | |
| --- | --- | --- | --- | --- | --- | --- | --- | --- |
|  | Women  % (95%CI) | Men  % (95%CI) | gap  p.p. |  | No education  % (95%CI) | Less than high school  % (95%CI) | High school or more  % (95%CI) | gap  p.p. |
| *Country (year)* |  |  |  |  |  |  |  |  |
| Algeria (2003) | 54.0 (52.0 - 56.0) | 44.5 (42.0 - 47.1) | 9.5 |  | 48.8 (46.5 - 51.1) | 51.0 (48.4 - 53.6) | 53.3 (49.3 - 57.3) | -4.5 |
| Algeria (2016) | 48.8 (47.2 - 50.5) | 50.6 (48.7 - 52.5) | -1.8 |  | 47.5 (44.4 - 50.6) | 55.2 (53.2 - 57.2) | 45.7 (43.8 - 47.6) | 1.8 |
| Benin (2008) | 79.0 (77.6 - 80.4) | 85.1 (83.8 - 86.3) | -6.1 |  | 85.3 (84.1 - 86.4) | 80.5 (78.5 - 82.4) | 68.4 (64.9 - 71.7) | 16.9 |
| Benin (2015) | 62.1 (59.7 - 64.5) | 69.4 (66.7 - 72.0) | -7.3 |  | 67.3 (64.8 - 69.8) | 66.6 (63.4 - 69.6) | 60.8 (56.4 - 65.0) | 6.5 |
| Botswana (2007) | 52.4 (50.0 - 54.7) | 58.1 (54.3 - 61.8) | -5.7 |  | 45.0 (38.5 - 51.7) | 54.8 (51.5 - 58.2) | 58.7 (55.9 - 61.6) | -13.7 |
| Botswana (2014) | 52.0 (49.2 - 54.8) | 53.8 (49.6 - 57.9) | -1.8 |  | 54.7 (48.5 - 60.7) | 55.5 (51.7 - 59.2) | 49.7 (45.7 - 53.7) | 5.0 |
| Cabo Verde (2007) | 61.5 (57.4 - 65.4) | 67.5 (62.0 - 72.6) | -6.0 |  | 58.1 (50.5 - 65.3) | 70.6 (66.5 - 74.5) | 57.3 (50.6 - 63.8) | 0.8 |
| Cabo Verde (2020) | 33.0 (30.5 - 35.5) | 41.7 (38.4 - 45.0) | -8.7 |  | 43.4 (35.6 - 51.4) | 48.1 (45.0 - 51.3) | 30.1 (27.4 - 33.1) | 13.3 |
| Eritrea (2004) | 16.1 (13.7 - 18.8) | 25.0 (21.8 - 28.5) | -8.9 |  | 17.2 (14.2 - 20.8) | 24.2 (20.3 - 28.6) | 19.3 (16.1 - 23.0) | -2.1 |
| Eritrea (2010) | 75.8 (74.3 - 77.2) | 81.9 (79.7 - 83.9) | -6.1 |  | 77.7 (76.0 - 79.3) | 77.6 (75.4 - 79.7) | 71.1 (66.7 - 75.1) | 6.6 |
| Eswatini (2007) | 54.9 (51.4 - 58.4) | 60.0 (55.0 - 64.7) | -5.1 |  | 59.5 (53.0 - 65.7) | 59.1 (54.6 - 63.4) | 54.1 (49.3 - 58.8) | 5.4 |
| Eswatini (2014) | 68.5 (65.6 - 71.4) | 71.6 (67.6 - 75.3) | -3.1 |  | 78.6 (72.7 - 83.6) | 75.6 (72.2 - 78.8) | 64.0 (60.3 - 67.6) | 14.6 |
| Malawi (2009) | 89.4 (88.2 - 90.6) | 90.6 (88.8 - 92.2) | -1.2 |  | 88.4 (86.0 - 90.5) | 91.6 (90.4 - 92.7) | 86.0 (82.6 - 88.8) | 2.4 |
| Malawi (2017) | 96.8 (95.6 - 97.7) | 96.4 (94.5 - 97.6) | 0.4 |  | 95.2 (89.8 - 97.8) | 97.7 (96.5 - 98.4) | 94.5 (91.9 - 96.4) | 0.7 |
| Mali (2007) | 12.8 (10.7 - 15.2) | 13.6 (11.0 - 16.8) | -0.8 |  | 15.3 (12.3 - 19.0) | 14.5 (11.8 - 17.6) | 8.3 (5.9 - 11.6) | 7.0 |
| Mali (2013) | 15.3 (12.9 - 18.0) | 14.7 (11.8 - 18.2) | 0.6 |  | 13.6 (10.9 - 16.9) | 18.3 (14.9 - 22.3) | 13.0 (9.7 - 17.2) | 0.6 |
| Central African Republic (2010) | 56.0 (52.9 - 59.2) | 60.0 (56.6 - 63.3) | -4.0 |  | 52.0 (45.9 - 57.9) | 61.9 (58.5 - 65.2) | 56.7 (53.0 - 60.2) | -4.7 |
| Central African Republic (2017) | 80.6 (78.4 - 82.5) | 83.2 (80.6 - 85.5) | -2.6 |  | 78.4 (73.9 - 82.3) | 83.0 (80.5 - 85.3) | 81.6 (79.1 - 84) | -3.2 |
| Sao Tome and Principe (2008) | 73.8 (71.3 - 76.1) | 82.2 (79.5 - 84.6) | -8.4 |  | 71.4 (65.1 - 77.0) | 81.0 (78.5 - 83.2) | 75.0 (72.0 - 77.8) | -3.6 |
| Sao Tome and Principe (2019) | 80.1 (77.5 - 82.5) | 74.4 (70.8 - 77.7) | 5.7 |  | 78.5 (69.5 - 85.4) | 79.4 (77.0 - 81.6) | 75.0 (69.9 - 79.4) | 3.5 |
| Zambia (2008) | 73.7 (70.8 - 76.4) | 56.1 (50.7 - 61.4) | 17.6 |  | 62.3 (51.1 - 72.4) | 74.4 (71.0 - 77.4) | 62.7 (58.3 - 66.9) | -0.4 |
| Zambia (2017) | 77.8 (75.5 - 79.9) | 82.4 (79.8 - 84.7) | -4.6 |  | 82.2 (77.4 - 86.2) | 84.3 (82.5 - 86.1) | 68.5 (64.2 - 72.4) | 13.7 |
| **Total wave 1** | 53.0 (39.2-66.8) | 54.6 (41.5 - 67.8) | -1.6 |  | 54.8 (40.1 - 69.5) | 60.4 (49.0 - 71.7) | 54.5 (42.2 - 66.9) | 0.3 |
| **Total wave 2** | 59.8 (42.1-77.6) | 63.0 (46.7 - 79.3) | -3.2 |  | 65.2 (51.5 - 78.9) | 67.4 (54.1 - 80.7) | 59.5 (42.5 - 76.4) | 5.7 |

**Supplementary Table 8**. Prevalence of occupational physical activity (≥150min/week).

|  | Gender | |  |  | Education | | | |
| --- | --- | --- | --- | --- | --- | --- | --- | --- |
|  | Women  % (95%CI) | Men  % (95%CI) | gap  p.p. |  | No education  % (95%CI) | Less than high school  % (95%CI) | High school or more  % (95%CI) | gap  p.p. |
| *Country (year)* |  |  |  |  |  |  |  |  |
| Algeria (2003) | 50.9 (48.8 - 52.9) | 42.7 (40.2 - 45.2) | 8.2 |  | 45.8 (43.6 - 48.1) | 48.8 (46.2 - 51.4) | 50.6 (46.6 - 54.6) | -4.8 |
| Algeria (2016) | 41.3 (39.7 - 43.0) | 45.4 (43.5 - 47.3) | -4.1 |  | 41.9 (38.8 - 45.0) | 49.3 (47.2 - 51.3) | 38.8 (37.0 - 40.7) | 3.1 |
| Benin (2008) | 77.3 (75.8 - 78.7) | 83.6 (82.2 - 84.9) | -6.3 |  | 84.0 (82.8 - 85.1) | 78.7 (76.6 - 80.7) | 65.3 (61.7 - 68.6) | 18.7 |
| Benin (2015) | 59.4 (56.9 - 61.8) | 68.0 (65.3 - 70.6) | -8.6 |  | 65.0 (62.4 - 67.5) | 64.4 (61.2 - 67.5) | 59.5 (55.1 - 63.8) | 5.5 |
| Botswana (2007) | 44.9 (42.5 - 47.3) | 49.4 (45.7 - 53.2) | -4.5 |  | 38.0 (31.7 - 44.7) | 48.5 (45.1 - 51.8) | 48.9 (46.0 - 51.8) | -10.9 |
| Botswana (2014) | 41.4 (38.6 - 44.3) | 47.4 (43.2 - 51.6) | -6.0 |  | 48.2 (42.0 - 54.4) | 47.9 (44.1 - 51.7) | 39.6 (35.8 - 43.7) | 8.6 |
| Cabo Verde (2007) | 58.8 (54.6 - 62.7) | 63.5 (57.9 - 68.8) | -4.7 |  | 56.4 (48.9 - 63.7) | 67.1 (62.8 - 71.1) | 53.8 (47.0 - 60.5) | 2.6 |
| Cabo Verde (2020) | 27.6 (25.2 - 30.0) | 36.8 (33.6 - 40.1) | -9.2 |  | 39.8 (32.3 - 47.8) | 44.1 (40.9 - 47.3) | 24.2 (21.7 - 27.0) | 15.6 |
| Eritrea (2004) | 15.4 (13.1 - 18.1) | 24.4 (21.2 - 27.9) | -9.0 |  | 16.8 (13.8 - 20.4) | 23.3 (19.4 - 27.6) | 18.7 (15.5 - 22.3) | -1.9 |
| Eritrea (2010) | 74.3 (72.7 - 75.7) | 81.1 (78.8 - 83.1) | -6.8 |  | 76.0 (74.2 - 77.7) | 76.6 (74.3 - 78.7) | 69.9 (65.5 - 74.0) | 6.1 |
| Eswatini (2007) | 49.3 (45.8 - 52.8) | 53.1 (48.2 - 58.0) | -3.8 |  | 54.0 (47.4 - 60.4) | 53.0 (48.4 - 57.4) | 47.6 (42.9 - 52.3) | 6.4 |
| Eswatini (2014) | 59.6 (56.4 - 62.7) | 65.1 (60.8 - 69.2) | -5.5 |  | 76.9 (70.9 - 82.0) | 68.3 (64.6 - 71.9) | 54.8 (51.0 - 58.6) | 22.1 |
| Malawi (2009) | 86.3 (84.9 - 87.6) | 89.0 (87.1 - 90.7) | -2.7 |  | 86.5 (83.9 - 88.7) | 89.6 (88.3 - 90.9) | 81.3 (77.6 - 84.5) | 5.2 |
| Malawi (2017) | 95.4 (94.0 - 96.6) | 94.6 (92.5 - 96.2) | 0.8 |  | 94.5 (89.3 - 97.2) | 96.3 (94.9 - 97.3) | 92.2 (89.1 - 94.5) | 2.3 |
| Mali (2007) | 11.0 (9.0 - 13.3) | 12.5 (10.0 - 15.6) | -1.5 |  | 13.7 (10.8 - 17.3) | 12.2 (9.8 - 15.1) | 8.1 (5.7 - 11.3) | 5.6 |
| Mali (2013) | 14.6 (12.3 - 17.3) | 13.5 (10.7 - 16.8) | 1.1 |  | 13.2 (10.5 - 16.5) | 16.9 (13.6 - 20.8) | 12.0 (8.8 - 16.1) | 1.2 |
| Central African Republic (2010) | 53.5 (50.3 - 56.6) | 55.2 (51.7 - 58.6) | -1.7 |  | 49.5 (43.5 - 55.5) | 58.1 (54.6 - 61.5) | 52.5 (48.9 - 56.1) | -3.0 |
| Central African Republic (2017) | 80.5 (78.4 - 82.4) | 83.2 (80.6 - 85.5) | -2.7 |  | 78.4 (73.9 - 82.3) | 82.9 (80.4 - 85.2) | 81.6 (79.1 - 84.0) | -3.2 |
| Sao Tome and Principe (2008) | 68.6 (66.0 - 71.1) | 79.8 (76.9 - 82.3) | -11.2 |  | 66.4 (60.0 - 72.4) | 77.4 (74.7 - 79.8) | 71.0 (67.9 - 73.9) | -4.6 |
| Sao Tome and Principe (2019) | 72.9 (70.0 - 75.5) | 68.1 (64.4 - 71.7) | 4.8 |  | 73.0 (63.5 - 80.7) | 72.2 (69.5 - 74.7) | 68.5 (63.2 - 73.3) | 4.5 |
| Zambia (2008) | 64.0 (60.9 - 67.0) | 45.7 (40.4 - 51.1) | 18.3 |  | 50.6 (39.6 - 61.6) | 65.0 (61.4 - 68.4) | 52.4 (47.9 - 56.8) | -1.8 |
| Zambia (2017) | 71.0 (68.6 - 73.4) | 75.7 (72.7 - 78.5) | -4.7 |  | 80.4 (75.3 - 84.6) | 78.6 (76.5 - 80.6) | 58.1 (53.5 - 62.5) | 22.3 |
| **Total wave 1** | 56.5 (42.8-70.1) | 58.3 (46.5-70.1) | -1.8 |  | 51.1 (35.7 - 66.5) | 56.6 (44.2-68.9) | 50.0 (37.6 - 62.4) | 1.5 |
| **Total wave 2** | 61.0 (45.0-76.9) | 64.2 (49.0-79.4) | -3.2 |  | 62.5 (47.3 - 77.6) | 63.4 (48.4-78.5) | 54.5 (36.4 - 72.5) | 8.0 |

**Supplementary Table 9**. Prevalence of some leisure-time physical activity (≥1min/week) according to age groups.

|  | 18-34 | 35-49 | 50+ | gap |
| --- | --- | --- | --- | --- |
|  | % (95%CI) | % (95%CI) | % (95%CI) | p.p. |
| Country |  |  |  |  |
| Algeria (2003) | 1.6 (1.1-2.5) | 1.1 (0.7-1.8) | 0.3 (0.1-1.0) | 1.3 |
| Algeria (2016) | 22.8 (21.0-24.7) | 10.7 (9.5-12.1) | 5.2 (4.2-6.4) | 17.6 |
| Benin (2008) | 13.3 (11.8-14.9) | 8.9 (7.8-10.2) | 3.6 (2.9-4.4) | 9.7 |
| Benin (2015) | 14.9 (12.9-17.2) | 5.8 (4.5-7.4) | 1.9 (1.1-3.1) | 13.0 |
| Botswana (2007) | 21.5 (18.9-24.3) | 11.3 (9.4-13.7) | 4.8 (2.7-8.2) | 16.7 |
| Botswana (2014) | 32.6 (29.2-36.2) | 20.1 (16.4-24.4) | 10.3 (7.4-14.2) | 22.3 |
| Cabo Verde (2007) | 30.5 (24.4-37.4) | 9.4 (7-12.7) | 5.7 (3.2-9.8) | 24.8 |
| Cabo Verde (2020) | 38.2 (34.9-41.6) | 22.5 (19.5-25.8) | 14.4 (11.9-17.2) | 23.8 |
| Eritrea (2004) | 4.7 (3.2-6.8) | 2.1 (1.1-4) | 2 (1.0-3.7) | 2.7 |
| Eritrea (2010) | 2.7 (2.0-3.7) | 0.9 (0.6-1.4) | 1.2 (0.8-1.8) | 1.5 |
| Eswatini (2007) | 24.3 (20.3-28.9) | 15.1 (12-18.9) | 12.4 (9.1-16.5) | 11.9 |
| Eswatini (2014) | 38.8 (35.2-42.5) | 16.8 (13.7-20.5) | 8.5 (5.5-12.8) | 30.3 |
| Malawi (2009) | 31.1 (28.7-33.6) | 19.2 (17-21.6) | 9.5 (7.6-12.0) | 21.6 |
| Malawi (2017) | 43.4 (37.6-49.4) | 26.8 (22.2-32.1) | 15.8 (10.8-22.5) | 27.6 |
| Mali (2007) | 4.6 (3.4-6.3) | 2.9 (1.5-5.4) | 2.9 (1.4-5.6) | 1.7 |
| Mali (2013) | 0.3 (0.0-1.8) | 0 (0-0) | 0.4 (0.1-2.7) | -0.1 |
| Central African Republic (2010) | 22.8 (19.8-26.2) | 15.9 (13.3-19) | 6.5 (4.5-9.4) | 16.3 |
| Central African Republic (2017) | 31.9 (28.5-35.6) | 20.3 (17.9-22.9) | 9.6 (7.6-12.1) | 22.3 |
| Sao Tome and Principe (2008) | 27.1 (24.3-30.1) | 16.4 (13.9-19.3) | 7.2 (5.1-10.1) | 19.9 |
| Sao Tome and Principe (2019) | 27.4 (24.4-30.5) | 15.4 (12.7-18.6) | 6.5 (4.4-9.4) | 20.9 |
| Zambia (2008) | 28.1 (24.8-31.6) | 23.9 (19.8-28.6) | 13.1 (9.5-17.9) | 15.0 |
| Zambia (2017) | 28.1 (25.5-30.9) | 11.4 (9.2-14) | 5 (3.1-7.9) | 23.1 |
| **Total wave 1** | 19.0 (13.8 - 24.3) | 11.4 (7.1 - 15.6) | 5.9 (3.5 - 8.3) | 13.1 |
| **Total wave 2** | 25.5 (18.1 - 33.0) | 15.1 (10.3 19.9) | 7.1 (4.9 - 9.3) | 18.4 |

**Supplementary Table 10**. Prevalence of leisure-time physical activity (≥150min/week) according to age groups.

|  | 18-34 | 35-49 | 50+ | gap |
| --- | --- | --- | --- | --- |
|  | % (95%CI) | % (95%CI) | % (95%CI) | p.p. |
| Country |  |  |  |  |
| Algeria (2003) | 1.1 (0.6-1.8) | 0.9 (0.6-1.5) | 0.3 (0.1-1.0) | 0.8 |
| Algeria (2016) | 15.6 (14.0-17.3) | 7.2 (6.2-8.3) | 3.3 (2.6-4.3) | 12.3 |
| Benin (2008) | 9.1 (7.9-10.5) | 6.3 (5.3-7.4) | 3.2 (2.6-4.1) | 5.9 |
| Benin (2015) | 9.5 (7.8-11.4) | 4.5 (3.3-6.0) | 1.6 (1.0-2.8) | 7.9 |
| Botswana (2007) | 17.1 (14.8-19.7) | 8.2 (6.6-10.1) | 3.7 (2.0-6.7) | 13.4 |
| Botswana (2014) | 25.5 (22.4-28.9) | 15.5 (12.1-19.8) | 6.7 (4.4-10.1) | 18.8 |
| Cabo Verde (2007) | 25.5 (19.6-32.5) | 6.8 (4.7-9.6) | 4.6 (2.5-8.4) | 20.9 |
| Cabo Verde (2020) | 31.2 (28.1-34.6) | 17.8 (15.1-20.8) | 12.3 (10.0-15.0) | 18.9 |
| Eritrea (2004) | 3.2 (2.0-5) | 1.6 (0.8-3.4) | 1.5 (0.7-3.2) | 1.7 |
| Eritrea (2010) | 2.0 (1.4-2.9) | 0.9 (0.6-1.3) | 1.1 (0.7-1.7) | 0.9 |
| Eswatini (2007) | 21.0 (17.2-25.4) | 10.6 (8.0-13.9) | 10.4 (7.5-14.4) | 10.6 |
| Eswatini (2014) | 32.4 (29.0-36.1) | 12.7 (9.9-16.1) | 6.0 (3.5-10.2) | 26.4 |
| Malawi (2009) | 22.0 (19.8-24.3) | 12.0 (10.2-14.0) | 6.1 (4.6-8.2) | 15.9 |
| Malawi (2017) | 31.8 (25.9-38.4) | 18.9 (14.9-23.8) | 10.8 (6.6-17.0) | 21 |
| Mali (2007) | 4.3 (3.1-5.9) | 2.5 (1.3-5.0) | 2.5 (1.2-5.2) | 1.8 |
| Mali (2013) | 0.3 (0.0-1.8) | 0.0 (0.0-0.0) | 0.4 (0.1-2.7) | -0.1 |
| Central African Republic (2010) | 17.1 (14.4-20.2) | 9.9 (7.9-12.3) | 4.6 (2.9-7.1) | 12.5 |
| Central African Republic (2017) | 21.2 (18.2-24.5) | 14.4 (12.3-16.7) | 7.5 (5.8-9.7) | 13.7 |
| Sao Tome and Principe (2008) | 20.3 (17.8-23) | 9.5 (7.5-11.8) | 4.8 (3.2-7.4) | 15.5 |
| Sao Tome and Principe (2019) | 17.9 (15.5-20.6) | 9.2 (7.1-11.8) | 4.3 (2.7-6.7) | 13.6 |
| Zambia (2008) | 20.5 (17.6-23.7) | 17.9 (14.3-22.1) | 11.2 (7.8-15.7) | 9.3 |
| Zambia (2017) | 21.2 (18.8-23.8) | 8.2 (6.4-10.5) | 4.4 (2.6-7.3) | 16.8 |
| **Total wave 1** | 14.6 (10.6 - 18.5) | 7.6 (5.3 - 10.0) | 4.3 (3.0 - 5.6) | 10.3 |
| **Total wave 2** | 19.0 (13.4 - 24.5) | 10.9 (7.7 - 14.1) | 5.3 (3.8 - 6.7) | 13.7 |

**Supplementary Table 11**. Prevalence of some transport physical activity (≥1min/week) according to age groups.

|  | 18-34 | 35-49 | 50+ | gap |
| --- | --- | --- | --- | --- |
|  | % (95%CI) | % (95%CI) | % (95%CI) | p.p. |
| Country |  |  |  |  |
| Algeria (2003) | 46.3 (43.6-49.1) | 47.4 (45.0 - 49.9) | 49.4 (46.2 - 52.6) | -3.1 |
| Algeria (2016) | 76.8 (75.0-78.5) | 74.4 (72.7 - 76.1) | 77.1 (75.1 - 79.0) | -0.3 |
| Benin (2008) | 94.1 (92.9-95.1) | 94.5 (93.5 - 95.4) | 92.4 (91.2 - 93.5) | 1.7 |
| Benin (2015) | 82.5 (80.4-84.4) | 79.7 (77.3 - 81.8) | 75.5 (71.8 - 78.8) | 7.0 |
| Botswana (2007) | 82.4 (79.9-84.6) | 79.4 (76.3 - 82.3) | 77.1 (72.6 - 81.0) | 5.3 |
| Botswana (2014) | 80.5 (77.6-83.1) | 75.4 (71.2 - 79.3) | 76.5 (71.7 - 80.7) | 4.0 |
| Cabo Verde (2007) | 89.0 (85.4-91.8) | 84.9 (81.4 - 87.8) | 78.9 (72.5 - 84.1) | 10.1 |
| Cabo Verde (2020) | 67.7 (64.5-70.8) | 70.8 (67.4 - 73.9) | 65.7 (62.0 - 69.1) | 2.0 |
| Eritrea (2004) | 82.7 (79.2-85.7) | 81.4 (77.5 - 84.8) | 77.7 (73.6 - 81.3) | 5.0 |
| Eritrea (2010) | 76.5 (74.2-78.7) | 79.5 (77.3 - 81.4) | 76.6 (74.6 - 78.4) | -0.1 |
| Eswatini (2007) | 64.4 (59.7-69.0) | 67.4 (62.8 - 71.6) | 58.6 (53.0 - 64.1) | 5.8 |
| Eswatini (2014) | 75.0 (72.0-77.9) | 74.6 (70.3 - 78.4) | 67.9 (60.8 - 74.3) | 7.1 |
| Malawi (2009) | 85.2 (83.3-86.9) | 82.2 (79.9 - 84.2) | 81.0 (78.0 - 83.6) | 4.2 |
| Malawi (2017) | 92.2 (89.8-94.1) | 89.7 (86.0 - 92.6) | 85.5 (80.5 - 89.4) | 6.7 |
| Mali (2007) | 59.0 (55.6-62.4) | 59.6 (54.0 - 64.8) | 57.9 (52.0 - 63.6) | 1.1 |
| Mali (2013) | 76.4 (72.0-80.3) | 73.8 (67.9 - 78.9) | 75.0 (69.4 - 79.9) | 1.4 |
| Central African Republic (2010) | 86.6 (83.7-89.1) | 88.0 (84.9 - 90.5) | 79.3 (75.3 - 82.7) | 7.3 |
| Central African Republic (2017) | 93.4 (91.3-95.0) | 92.7 (91.1 - 94.1) | 85.2 (82.5 - 87.6) | 8.2 |
| Sao Tome and Principe (2008) | 76.3 (73.5-79.0) | 78.0 (75.0 - 80.8) | 73.4 (69.1 - 77.3) | 2.9 |
| Sao Tome and Principe (2019) | 60.8 (57.2-64.3) | 56.8 (52.6 - 60.9) | 55.2 (49.7 - 60.6) | 5.6 |
| Zambia (2008) | 93.0 (90.8-94.7) | 93.4 (90.4 - 95.5) | 88.4 (83.9 - 91.9) | 4.6 |
| Zambia (2017) | 85.8 (83.7-87.7) | 84.9 (82.0 - 87.3) | 82.7 (78.8 - 86.0) | 3.1 |
| **Total wave 1** | 79.2 (77.1 - 81.2) | 78.6 (76.1 - 81.2) | 75.2 (72.7 - 77.7) | 4.n |
| **Total wave 2** | 78.2 (74.1 - 83.7) | 77.5 (72.8 - 82.3) | 74.6 (71.6 - 77.6) | 3.6 |

**Supplementary Table 12**. Prevalence of transport physical activity (≥150min/week) according to age groups.

|  | 18-34 | 35-49 | 50+ | gap |
| --- | --- | --- | --- | --- |
|  | % (95%CI) | % (95%CI) | % (95%CI) | p.p. |
| Country |  |  |  |  |
| Algeria (2003) | 30.9 (28.5 - 33.5) | 33.2 (31.0 - 35.6) | 35.0 (32.0 - 38.1) | -4.1 |
| Algeria (2016) | 54.8 (52.7 - 56.9) | 50.5 (48.5 - 52.5) | 54.8 (52.4 - 57.1) | 0 |
| Benin (2008) | 84.2 (82.5 - 85.8) | 86.0 (84.4 - 87.4) | 80.9 (79.2 - 82.5) | 3.3 |
| Benin (2015) | 62.6 (59.9 - 65.2) | 57.7 (54.7 - 60.6) | 56.5 (52.3 - 60.5) | 6.1 |
| Botswana (2007) | 45.3 (42.2 - 48.4) | 42.6 (39.1 - 46.1) | 46.9 (42.1 - 51.7) | -1.6 |
| Botswana (2014) | 50.9 (47.4 - 54.5) | 50.8 (46.1 - 55.4) | 53.2 (48.1 - 58.3) | -2.3 |
| Cabo Verde (2007) | 49.6 (43.2 - 56.0) | 57.9 (53.2 - 62.5) | 55.6 (49.1 - 61.9) | -6 |
| Cabo Verde (2020) | 32.2 (29.2 - 35.5) | 37.4 (33.9 - 41.1) | 38.5 (34.8 - 42.3) | -6.3 |
| Eritrea (2004) | 59.6 (55.4 - 63.7) | 59.2 (54.5 - 63.7) | 55.8 (51.2 - 60.3) | 3.8 |
| Eritrea (2010) | 60.6 (58.0 - 63.1) | 63.4 (60.9 - 65.8) | 61.4 (59.1 - 63.6) | -0.8 |
| Eswatini (2007) | 39.3 (34.6 - 44.1) | 44.6 (40.0 - 49.2) | 40.4 (35.0 - 46.1) | -1.1 |
| Eswatini (2014) | 43.3 (39.7 - 46.9) | 47.4 (42.6 - 52.2) | 37.0 (31.3 - 43.0) | 6.3 |
| Malawi (2009) | 55.8 (53.2 - 58.3) | 55.5 (52.6 - 58.2) | 50.7 (46.9 - 54.4) | 5.1 |
| Malawi (2017) | 74.2 (69.7 - 78.2) | 71.2 (66.2 - 75.8) | 64.1 (57.9 - 69.9) | 10.1 |
| Mali (2007) | 35.7 (32.5 - 39.1) | 36.9 (31.8 - 42.4) | 39.9 (34.3 - 45.8) | -4.2 |
| Mali (2013) | 62.1 (57.2 - 66.7) | 60.2 (54.0 - 66.2) | 58.5 (52.4 - 64.3) | 3.6 |
| Central African Republic (2010) | 78.3 (75.1 - 81.3) | 78.3 (74.9 - 81.4) | 65.8 (61.4 - 70.0) | 12.5 |
| Central African Republic (2017) | 89.1 (86.6 - 91.2) | 87.2 (85.2 - 89.0) | 76.6 (73.5 - 79.5) | 12.5 |
| Sao Tome and Principe (2008) | 49.8 (46.5 - 53.0) | 53.9 (50.4 - 57.3) | 44.6 (39.9 - 49.4) | 5.2 |
| Sao Tome and Principe (2019) | 30.6 (27.5 - 33.9) | 29.0 (25.4 - 32.8) | 26.5 (22.0 - 31.5) | 4.1 |
| Zambia (2008) | 47.5 (43.7 - 51.3) | 49.7 (44.6 - 54.8) | 39.0 (33.2 - 45.2) | 8.5 |
| Zambia (2017) | 58.5 (55.5 - 61.4) | 59.7 (56.0 - 63.3) | 55.4 (50.6 - 60.1) | 3.1 |
| **Total wave 1** | 52.4 (45.5 - 59.4) | 54.4 (47.4 - 61.5) | 50.8 (45.5 - 56.0) | 1.6 |
| **Total wave 2** | 56.2 (50.1 - 62.3) | 55.8 (49.6 - 62.0) | 52.5 (48.8 - 56.2) | 3.7 |

**Supplementary Table 13**. Prevalence of some occupational physical activity (≥1min/week) according to age groups.

|  | 18-34 | 35-49 | 50+ | gap |
| --- | --- | --- | --- | --- |
|  | % (95%CI) | % (95%CI) | % (95%CI) | p.p. |
| Country |  |  |  |  |
| Algeria (2003) | 42.4 (39.3 - 45.6) | 52.8 (50.4 - 55.2) | 53.0 (50.2 - 55.7) | -10.6 |
| Algeria (2016) | 40.3 (38.0 - 42.6) | 56.5 (54.5 - 58.5) | 48.9 (46.8 - 51.1) | -8.6 |
| Benin (2008) | 76.2 (74.3 - 77.9) | 86.6 (85.1 - 88.0) | 83.7 (82.0 - 85.3) | -7.5 |
| Benin (2015) | 59.6 (55.4 - 63.6) | 68.2 (65.3 - 70.9) | 66.2 (63.5 - 68.7) | -6.6 |
| Botswana (2007) | 48.1 (43.3 - 53.0) | 57.6 (53.9 - 61.1) | 56.8 (53.8 - 59.9) | -8.7 |
| Botswana (2014) | 53.6 (48.4 - 58.6) | 54.6 (49.9 - 59.2) | 52.1 (48.6 - 55.6) | 1.5 |
| Cabo Verde (2007) | 45.5 (39.3 - 51.9) | 66.3 (61.8 - 70.4) | 69.4 (63.6 - 74.7) | -23.9 |
| Cabo Verde (2020) | 36.7 (33.2 - 40.5) | 40.1 (36.4 - 43.8) | 35.9 (32.7 - 39.2) | 0.8 |
| Eritrea (2004) | 16.4 (13.3 - 20.1) | 23.9 (20.1 - 28.2) | 19.9 (16.8 - 23.5) | -3.5 |
| Eritrea (2010) | 71.6 (69.4 - 73.6) | 80.2 (78.1 - 82.1) | 76.4 (74.1 - 78.7) | -4.8 |
| Eswatini (2007) | 49.5 (43.9 - 55.2) | 62.8 (58.2 - 67.2) | 55.8 (51.0 - 60.6) | -6.3 |
| Eswatini (2014) | 72.5 (66.9 - 77.4) | 70.8 (66.4 - 74.8) | 68.9 (65.6 - 72.0) | 3.6 |
| Malawi (2009) | 89 (86.5 - 91.2) | 90.3 (88.4 - 91.8) | 90.3 (88.6 - 91.7) | -1.3 |
| Malawi (2017) | 93.7 (90.1 - 96.1) | 96.2 (93.6 - 97.8) | 97.5 (96.4 - 98.3) | -3.8 |
| Mali (2007) | 15.8 (12.0 - 20.6) | 14.3 (10.9 - 18.7) | 11.7 (9.6 - 14.1) | 4.1 |
| Mali (2013) | 11.2 (7.9 - 15.6) | 13.1 (9.4 - 18.0) | 17.6 (14.2 - 21.6) | -6.4 |
| Central African Republic (2010) | 49 (44.5 - 53.5) | 60.1 (56.2 - 63.9) | 60.8 (57.0 - 64.4) | -11.8 |
| Central African Republic (2017) | 68.6 (65.3 - 71.8) | 83.2 (80.9 - 85.2) | 83.5 (80.6 - 86.1) | -14.9 |
| Sao Tome and Principe (2008) | 69.3 (64.8 - 73.4) | 80.4 (77.5 - 83.0) | 79.4 (76.6 - 81.9) | -10.1 |
| Sao Tome and Principe (2019) | 74.2 (69.1 - 78.7) | 78.6 (74.9 - 81.9) | 79.8 (76.7 - 82.5) | -5.6 |
| Zambia (2008) | 53.4 (47.2 - 59.5) | 73.9 (69.1 - 78.2) | 72.7 (69.2 - 76.0) | -19.3 |
| Zambia (2017) | 78.5 (74.2 - 82.3) | 80.0 (76.8 - 82.8) | 80.3 (78.0 - 82.5) | -1.8 |
| **Total wave 1** | 59.4 (47.9 - 70.9) | 60.9 (49.2 - 72.5) | 50.4 (33.1 - 67.8) | 9 |
| **Total wave 2** | 64.3 (48.3 - 80.3) | 65.6 (52.5 - 78.7) | 60.0 (42.4 - 77.7) | 4.3 |

**Supplementary Table 14**. Prevalence of occupational physical activity (≥150min/week) according to age groups.

|  | 18-34 | 35-49 | 50+ | gap |
| --- | --- | --- | --- | --- |
|  | % (95%CI) | % (95%CI) | % (95%CI) | p.p. |
| Country |  |  |  |  |
| Algeria (2003) | 50.7 (48.0 - 53.4) | 50.2 (47.7 - 52.6) | 39.3 (36.2 - 42.5) | 11.4 |
| Algeria (2016) | 42.2 (40.1 - 44.3) | 50.4 (48.4 - 52.4) | 34.4 (32.2 - 36.6) | 7.8 |
| Benin (2008) | 82.3 (80.5 - 84.0) | 85.1 (83.5 - 86.5) | 74.3 (72.4 - 76.1) | 8 |
| Benin (2015) | 63.8 (61.1 - 66.4) | 67.0 (64.2 - 69.8) | 57.2 (53.0 - 61.3) | 6.6 |
| Botswana (2007) | 47.0 (44.0 - 50.1) | 49.7 (46.1 - 53.3) | 42.7 (38.0 - 47.5) | 4.3 |
| Botswana (2014) | 42.2 (38.7 - 45.8) | 49.0 (44.3 - 53.6) | 45.9 (40.8 - 51.1) | -3.7 |
| Cabo Verde (2007) | 66.3 (60.3 - 71.8) | 62.9 (58.4 - 67.2) | 41.7 (35.7 - 47.9) | 24.6 |
| Cabo Verde (2020) | 29.1 (26.1 - 32.3) | 36.6 (33.1 - 40.3) | 33.0 (29.5 - 36.7) | -3.9 |
| Eritrea (2004) | 19.4 (16.2 - 22.9) | 23.0 (19.2 - 27.2) | 16.0 (12.9 - 19.6) | 3.4 |
| Eritrea (2010) | 74.5 (72.1 - 76.8) | 79.1 (76.9 - 81.1) | 70.1 (67.8 - 72.2) | 4.4 |
| Eswatini (2007) | 49.3 (44.4 - 54.1) | 57.5 (52.9 - 62.1) | 42.5 (36.9 - 48.2) | 6.8 |
| Eswatini (2014) | 59.0 (55.4 - 62.5) | 65.5 (60.8 - 69.9) | 68.6 (62.9 - 73.8) | -9.6 |
| Malawi (2009) | 87.8 (86.0 - 89.3) | 87.6 (85.6 - 89.4) | 87.2 (84.6 - 89.5) | 0.6 |
| Malawi (2017) | 96.3 (94.9 - 97.3) | 94.5 (91.6 - 96.4) | 91.3 (87.3 - 94.1) | 5 |
| Mali (2007) | 10.4 (8.5 - 12.7) | 12.1 (8.9 - 16.2) | 14.4 (10.7 - 19.0) | -4 |
| Mali (2013) | 16.8 (13.5 - 20.8) | 13.1 (9.4 - 18.0) | 10.0 (6.9 - 14.3) | 6.8 |
| Central African Republic (2010) | 55.9 (52.1 - 59.6) | 57.2 (53.3 - 61.1) | 46.3 (41.9 - 50.8) | 9.6 |
| Central African Republic (2017) | 83.5 (80.6 - 86.1) | 83.1 (80.9 - 85.2) | 68.6 (65.3 - 71.8) | 14.9 |
| Sao Tome and Principe (2008) | 75.0 (72.0 - 77.7) | 77.0 (74.0 - 79.8) | 65.4 (60.8 - 69.7) | 9.6 |
| Sao Tome and Principe (2019) | 72.6 (69.2 - 75.7) | 72.6 (68.6 - 76.2) | 66.1 (60.7 - 71.1) | 6.5 |
| Zambia (2008) | 63.0 (59.2 - 66.6) | 64.3 (59.2 - 69.0) | 42.6 (36.7 - 48.8) | 20.4 |
| Zambia (2017) | 73.0 (70.3 - 75.5) | 73.5 (70.1 - 76.8) | 74.0 (69.5 - 78.1) | -1 |
| **Total wave 1** | 55.2 (42.6 - 67.8) | 57.0 (44.7 - 69.3) | 46.6 (28.5 - 64.7) | 8.6 |
| **Total wave 2** | 59.4 (41.3 - 77.5) | 62.3 (47.9 - 76.6) | 56.3 (37.6 - 74.9) | 3.1 |

**Supplementary Table 15**. Prevalence of meeting the WHO recommendations of physical activity*.

|  | 18-34 | 35-49 | 50+ | gap |
| --- | --- | --- | --- | --- |
|  | % (95%CI) | % (95%CI) | % (95%CI) | p.p. |
| Country |  |  |  |  |
| Algeria (2003) | 66.9 (64.2 - 69.4) | 66.1 (63.7 - 68.4) | 61.0 (57.9 - 64.1) | 5.9 |
| Algeria (2016) | 78.0 (76.2 - 79.6) | 76.4 (74.7 - 78.0) | 70.4 (68.2 - 72.4) | 7.6 |
| Benin (2008) | 94.6 (93.5 - 95.6) | 95.8 (94.8 - 96.5) | 90.6 (89.3 - 91.8) | 4 |
| Benin (2015) | 84.8 (82.8 - 86.7) | 83.2 (80.9 - 85.2) | 77.0 (73.5 - 80.1) | 7.8 |
| Botswana (2007) | 76.7 (74.0 - 79.1) | 72.7 (69.2 - 75.9) | 68.9 (64.2 - 73.2) | 7.8 |
| Botswana (2014) | 80.0 (77.4 - 82.3) | 75.5 (71.0 - 79.5) | 76.5 (72.2 - 80.3) | 3.5 |
| Cabo Verde (2007) | 85.0 (81.0 - 88.3) | 80.3 (76.6 - 83.5) | 65.1 (58.6 - 71.1) | 19.9 |
| Cabo Verde (2020) | 67.2 (64.0 - 70.2) | 64.8 (61.2 - 68.3) | 59.4 (55.7 - 63.0) | 7.8 |
| Eritrea (2004) | 67.4 (63.3 - 71.2) | 65.0 (60.3 - 69.3) | 60.8 (56.3 - 65.2) | 6.6 |
| Eritrea (2010) | 87.2 (85.3 - 88.8) | 89.1 (87.5 - 90.6) | 84.5 (82.8 - 86.1) | 2.7 |
| Eswatini (2007) | 69.2 (64.6 - 73.5) | 75.1 (70.9 - 78.8) | 61.6 (55.9 - 66.9) | 7.6 |
| Eswatini (2014) | 82.2 (79.5 - 84.6) | 82.7 (79.0 - 85.9) | 78.2 (73.3 - 82.4) | 4 |
| Malawi (2009) | 94.6 (93.4 - 95.6) | 94.3 (92.8 - 95.4) | 93.2 (91.2 - 94.8) | 1.4 |
| Malawi (2017) | 98.8 (97.9 - 99.4) | 98.8 (97.7 - 99.4) | 95.8 (93.4 - 97.3) | 3 |
| Mali (2007) | 43.1 (39.7 - 46.6) | 43.0 (37.6 - 48.5) | 43.9 (38.2 - 49.8) | -0.8 |
| Mali (2013) | 66.3 (61.5 - 70.8) | 64.8 (58.6 - 70.5) | 61.9 (55.9 - 67.6) | 4.4 |
| Central African Republic (2010) | 84.5 (81.5 - 87.1) | 84.7 (81.5 - 87.5) | 74.2 (70.1 - 78.0) | 10.3 |
| Central African Republic (2017) | 96.9 (95.5 - 97.9) | 95.3 (93.9 - 96.3) | 84.4 (81.6 - 86.8) | 12.5 |
| Sao Tome and Principe (2008) | 88.6 (86.3 - 90.5) | 88.9 (86.6 - 90.9) | 77.3 (73.2 - 81.0) | 11.3 |
| Sao Tome and Principe (2019) | 84.9 (82.0 - 87.4) | 81.0 (77.4 - 84.1) | 73.2 (68.0 - 77.8) | 11.7 |
| Zambia (2008) | 88.2 (85.5 - 90.4) | 85.4 (81.4 - 88.7) | 70.5 (64.6 - 75.8) | 17.7 |
| Zambia (2017) | 89.3 (87.5 - 90.9) | 90.2 (87.9 - 92.1) | 86.0 (82.5 - 88.8) | 3.3 |
| **Total wave 1** | 78.3 (73.4 - 83.1) | 77.7 (71.9 - 83.5) | 69.8 (60.1 - 79.5) | 8.5 |
| **Total wave 2** | 78.5 (71.0 - 86.0) | 82.1 (74.6 - 89.5) | 77.1 (68.8 - 85.3) | 1.4 |

Note. *Using WHO 2020 guidelines on physical activity of at least 75min/week of vigorous physical activity or 150min/week of the combination of moderate and vigorous physical activity.
